# Unraveling the genetics of arsenic toxicity with cellular morphology QTL

**DOI:** 10.1371/journal.pgen.1011248

**Published:** 2024-04-25

**Authors:** Callan O’Connor, Gregory R. Keele, Whitney Martin, Timothy Stodola, Daniel Gatti, Brian R. Hoffman, Ron Korstanje, Gary A. Churchill, Laura G. Reinholdt

**Affiliations:** 1 The Jackson Laboratory, Bar Harbor, Maine, United States of America; 2 Graduate School of Biomedical Sciences, Tufts University, Boston, Massachusetts, United States of America; 3 RTI International, Research Triangle Park, Durham, North Carolina, United States of America; National Institutes of Health, UNITED STATES

## Abstract

The health risks that arise from environmental exposures vary widely within and across human populations, and these differences are largely determined by genetic variation and gene-by-environment (gene–environment) interactions. However, risk assessment in laboratory mice typically involves isogenic strains and therefore, does not account for these known genetic effects. In this context, genetically heterogenous cell lines from laboratory mice are promising tools for population-based screening because they provide a way to introduce genetic variation in risk assessment without increasing animal use. Cell lines from genetic reference populations of laboratory mice offer genetic diversity, power for genetic mapping, and potentially, predictive value for *in vivo* experimentation in genetically matched individuals. To explore this further, we derived a panel of fibroblast lines from a genetic reference population of laboratory mice (the Diversity Outbred, DO). We then used high-content imaging to capture hundreds of cell morphology traits in cells exposed to the oxidative stress-inducing arsenic metabolite monomethylarsonous acid (MMA^III^). We employed dose-response modeling to capture latent parameters of response and we then used these parameters to identify several hundred cell morphology quantitative trait loci (cmQTL). Response cmQTL encompass genes with established associations with cellular responses to arsenic exposure, including *Abcc4* and *Txnrd1*, as well as novel gene candidates like *Xrcc2*. Moreover, baseline trait cmQTL highlight the influence of natural variation on fundamental aspects of nuclear morphology. We show that the natural variants influencing response include both coding and non-coding variation, and that cmQTL haplotypes can be used to predict response in orthogonal cell lines. Our study sheds light on the major molecular initiating events of oxidative stress that are under genetic regulation, including the NRF2-mediated antioxidant response, cellular detoxification pathways, DNA damage repair response, and cell death trajectories.

## Author summary

Exposure to environmental toxicants leads to adverse health outcomes. Natural genetic variation regulates the likelihood and severity of these outcomes, but studying the underlying genes and pathways in human populations is challenging. Population-based rodent models simulate the genetic variation of the human population offering numerous advantages as experimental genetic models for studying gene–environment interactions and in chemical risk asessment. These include exquisite environmental control and higher power for mapping the genes and pathways that influence sensitivity and resilience to environmental exposures. We leveraged a genetically diverse laboratory mouse population to investigate the genetic regulation of arsenic response. To minimize animal use, cells were derived from each individual animal through a minimally invasive tail biopsy. These cells provide a reusable genetic resource for chemical or drug screening through which predictions of risk can be made and tested in genetically matched laboratory mice. To evaluate the utility of this resource, we used high content imaging to quantify changes in cell morphology following exposure to the arsenic metabolite MMA^III^. Using dose-response modeling, we identified the subset of morphological changes that are informative of arsenic response and that exhibit robust interindividual variation. We then used genetic association mapping to identify several hundred loci regulating individual differences in arsenic response. To nominate candidate driver genes, we integrated various lines of evidence from published arsenic studies and molecular data from the same mouse population. Our work demonstrates that genetic variation in the molecular pathways regulating arsenic transport, oxidative stress, and DNA damage contribute to variation in arsenic response. Additionally, we show that this genetic variation predicts cellular response in independent experiments. Our data establish a population-based approach for studying gene–environment interactions using inexpensive and high-throughput cell morphology traits in an experimental system that enables recursive *in vitro* and *in vivo* experimentation across genetically matched individuals.

## Introduction

Recent advances in microscopy-based, high-content cellular screening (HCS) have made it cost-effective to analyze cellular phenotypes at scale [1–5]. Cellular morphology is a useful phenotype for understanding how genetic factors regulate the state of metazoan cells, ranging from yeast to human induced pluripotent stem cells (iPSCs) [6,7]. The established correlation of cell morphology traits to molecular -omics traits underscores the potential to quantitatively analyze morphological changes as an indicator of cell state changes in response to environmental perturbations [8–10]. Here, we use cell morphology traits acquired from HCS to identify genomic loci associated with variation in the cellular response to acute arsenical exposure.

Arsenic is a known carcinogen and a widespread contaminant of groundwater, exposing up to an estimated 220 million people worldwide [11]. At the cellular level, arsenic exposure induces oxidative stress, DNA damage, and cytotoxicity [12–16]. Ingested inorganic arsenic is metabolized by the liver through methylation and reducing reactions that generate circulating metabolites including monomethylarsonic acid (MMA^V^), monomethylarsonous acid (MMA^III^), dimethylarsinic acid (DMA^V^), and dimethylarsinous acid (DMA^III^) [17–19]. Genetic mapping studies have revealed genes and variants that regulate arsenic metabolism, as well as oxidative stress response and DNA damage repair [20–36]. The metabolite MMA^III^ is more toxic than ingested inorganic arsenic and causes DNA damage through oxidative stress, although not all cell types, such as fibroblasts, can methylate arsenic [16,37,38,39]. We sought to harness a population-based cellular model from laboratory mice and uncover gene–environment interactions for the metabolite MMA^III^ using high-content cell morphology traits.

Genetically diverse laboratory mouse populations are powerful experimental tools for genetic analysis, and they are well established in the study of gene–environment interactions *in vivo* [40,41]. Cell lines from these genetic reference populations offer potential as a ‘new approach methodology’ wherein genetic screens can be performed *in vitro* to identify haplotypes that confer sensitivity and resilience to toxicant exposure [42,43]. Where informative cellular or molecular phenotypes exist, approaches such as these have the potential to reduce the use of animals in assessing hazards of chemical exposure. Thus, we created a genetically diverse panel of primary fibroblast cell lines from the Diversity Outbred (DO) mouse population [44]. DO mice are outbred animals descended from eight inbred mouse strains: A/J (AJ), C57BL/6J (B6), 129S1/SvImJ (129), NOD/ShiLtJ (NOD), NZO/HILtJ (NZO), CAST/EiJ (CAST), PWK/PhJ (PWK), and WSB/EiJ (WSB). These inbred strains represent three sub-species of *Mus musculus* and thus possess far more genetic variation than traditional mouse crosses, capturing roughly 45 million segregating single nucleotide polymorphisms (SNPs) [44,45].

Using a HCS technique similar to Cell Painting where cells and organelles are stained with multiplexed fluorophores [1], we show that changes in cell morphology that occur during an acute oxidative stress response can be summarized through dose-response modeling. These cell state changes vary across genetically diverse fibroblast lines, revealing both sensitivity and resiliency to arsenic exposure. We used the inorganic arsenic metabolite MMA^III^ for our screen because MMA^III^ induces cancer in organs that are downstream of the liver, such as the kidney [46,47]. Using quantitative trait locus (QTL) mapping, we found 854 suggestive cell morphology QTLs (cmQTLs; LOD score > 7.5) that regulate the cellular response to MMA^III^. Additionally, we show that the effects of the cmQTLs are both reproducible and predictive of a cell line’s MMA^III^ sensitivity. At the gene and pathway level, many cmQTLs recapitulate genetic associations that have been previously found in human population studies, demonstrating the translational utility of our population-based cellular model. We highlight the roles of *Xrcc2 and Txnrd1* alleles that modulate MMA^III^-induced cell death, and we provide new associations for a host of candidate genes that interact with MMA^III^. This proof-of-concept study demonstrates that high throughput cell morphology traits provide robust phenotypes for population-based screening of gene–environment interactions in the context of a chemical exposure. Cell lines from laboratory mouse genetic reference populations provide an avenue to introduce genetic variation in risk assessment, where susceptible and resilient genetic backgrounds can be identified *in vitro* for targeted *in vivo* studies.

## Results

**Fig 1:**
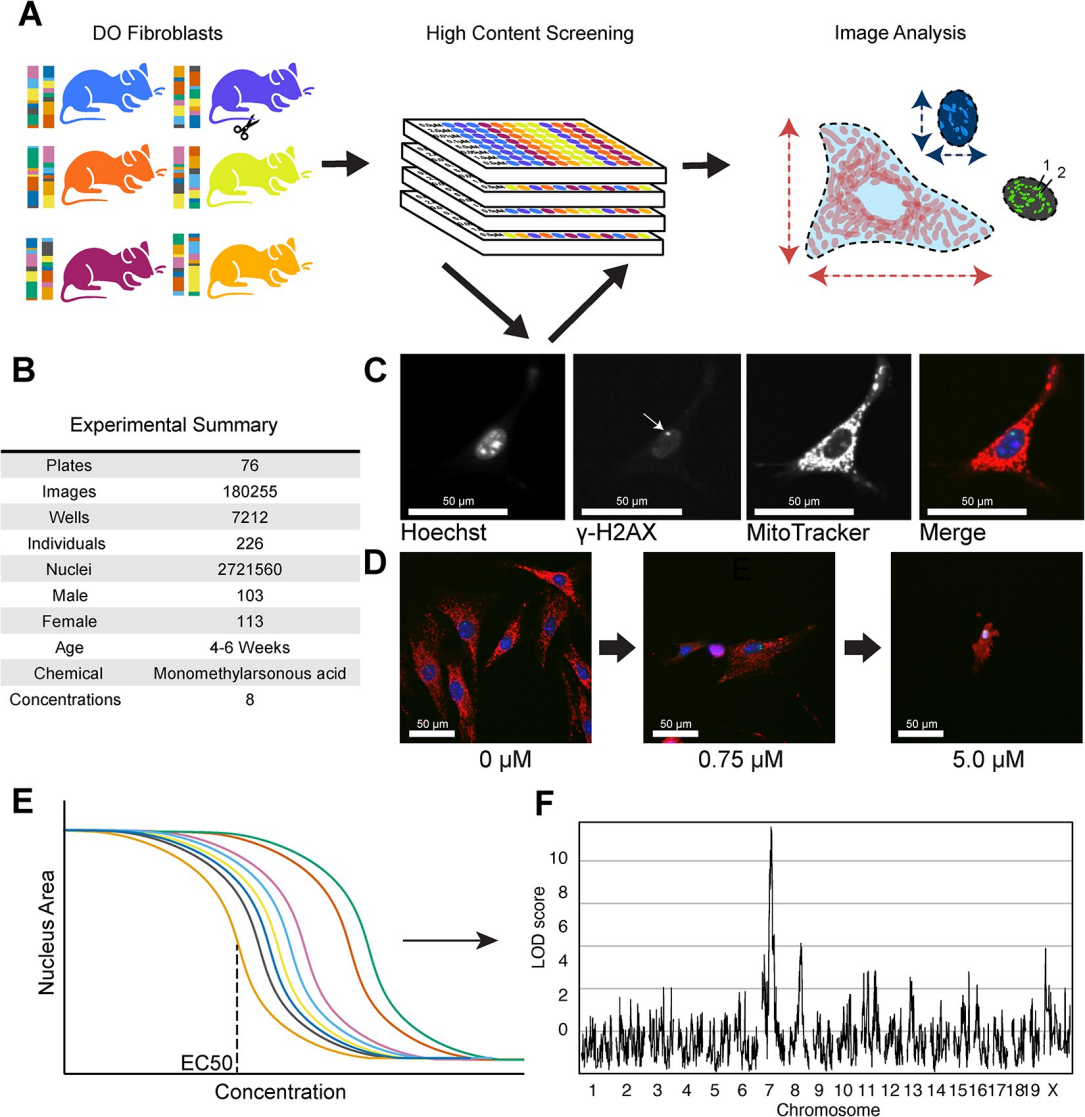
HCS of MMA^III^-exposed DO Fibroblasts. (A) 600+ primary fibroblasts were derived from Diversity Outbred (DO) mice aged 4–6 weeks. 226 of these DO fibroblast lines were exposed to 8 concentrations of MMA^III^ (0 μM, 0.01 μM, 0.1 μM, 0.75 μM, 1.0 μM, 1.25 μM, 2.0 μM, and 5.0 μM). Cell lines were randomly seeded into 96-well plates (4 columns spanning two plates, see Supplementals for more information). Image analysis was performed at the whole well level and summarized across concentrations using dose-response modeling. (B) Table with experimental summary. (C) Example images showing fibroblasts labeled with Hoechst 33342, an anti-gamma γH2AX antibody with an Alexafluor 488 donkey anti-rabbit secondary, MitoTracker Deep Red, and the merged image. Plates were imaged using an Operetta High Content Imager (PerkinElmer) at 20X. (D) Example merged images showing a fibroblast morphology across three representative doses of MMA^III^ (0 μM, 0.75 μM, 5.0 μM). (E) Dose-response modeling was performed to summarize the cellular morphological changes across all 8 concentrations of MMA^III^. Model parameters describing the starting asymptote, shape (slope), sensitivity (EC5, EC10, EC25, EC50, EC75, EC90), and maximum asymptote were extracted from these models as quantitative traits. (F) Quantitative trait loci (QTL) mapping was used to associate variation in the dose-response model parameters (i.e., EC50) with genetic variation in the DO fibroblast population through whole genome QTL scans.

Cellular morphology is influenced by genetic variation and environmental factors such as chemical exposures [7,48]. We sought to use morphological traits to quantify the key cellular events in response to MMA^III^ exposure and to identify the genetic determinants of MMA^III^ sensitivity in an unbiased screen. We established a population-based cellular model by deriving a panel of ‘tail tip’ fibroblast lines from each of 600 mice Diversity Outbred (DO) mice (**Fig 1A and 1B**). Tail tip fibroblast cell lines can be readily established through minimally invasive biopsies, the cells are adherent, and they can be easily maintained for many passages when collected from young donors. To observe effects of acute exposure to MMA^III^, we treated 226 of these DO fibroblast lines with eight increasing concentrations of monomethylarsonous acid (MMA^III^) across 76 randomized 96-well plates (**Fig 1D**; see Methods). Based on the genetic architecture of the DO population, we expected 226 individual cell lines would allow us to detect QTLs explaining >20% of the phenotypic variance with 90% power [49,50]. We employed multiplex fluorescent labeling to quantify changes in cell morphology traits associated with oxidative stress and genotoxicity. These included nuclei (Hoechst 33342), mitochondria (MitoTracker Deep Red), and to quantify DNA damage indirect immunolabeling with an antibody recognizing the DNA damage response marker, phosphorylated H2AX (γH2AX)[51,52] (**Fig 1C**). We captured 180,255 images and performed image analysis using Harmony 4.9 to extract 673 image-based, morphological traits from 2,721,560 cells (**Fig 1B)**.

### Sources of variation in cell morphology traits

**Fig 2:**
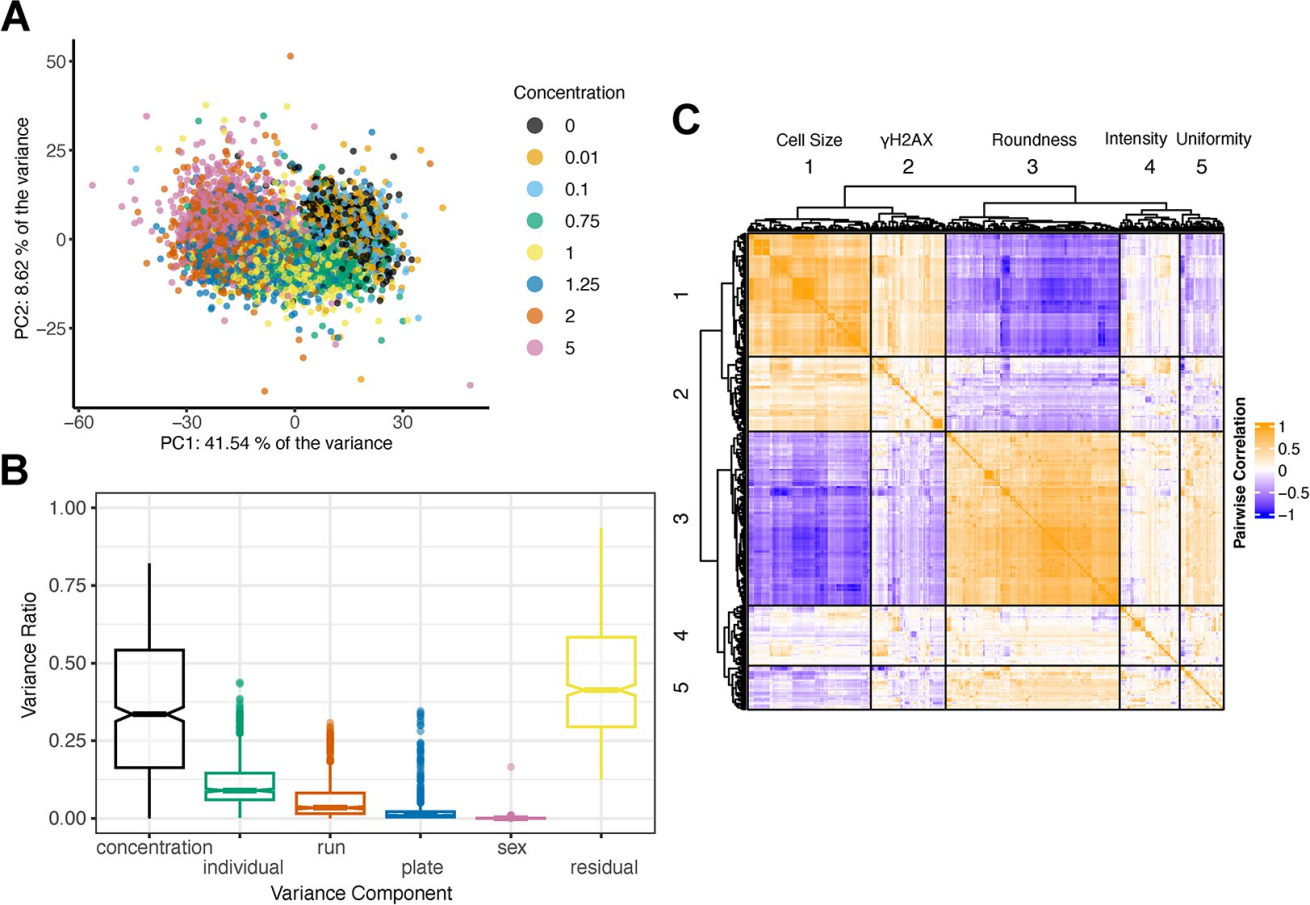
HCS Features are Influenced by MMA^III^ Concentration and Genetic Background. (A) Principal Component Analysis (PCA) of the raw image analysis feature dataset colored by the concentration of MMA^III^. Among known factors, MMA^III^ concentration was correlated with PC1 (41.54%). (B) Boxplot showing the aggregated results from variance component analysis (VCA) performed across all cellular features, including MMA^III^ concentration, DO cell lines (individual), each 96-well plate (plate), run (group batch), sex, and residual variation. (C) Heatmap showing the Pearson’s pairwise correlation structure of the raw cellular features. The heatmap and dendrogram were generated using the R package ComplexHeatmap’s Heatmap() function with column_split and row_split, each set to 5.

We assessed the main drivers of variation in these morphological traits by performing principal components analysis. The first principal component, accounting for 41.5% of the observed variation across all morphological traits were correlated with MMA^III^ concentration, and there was a clear dose-dependent effect (**Fig 2A**). Following Matthew et al. [7], we performed a decomposition of the sources of variation contributing to each trait by fitting a random effects linear model with terms for inter-plate effects (‘plate’), batch effects (12 samples per ‘run’), MMA^III^ concentration (‘concentration’), DO donor (‘individual’), and the sex of cell donor (‘sex’) (**Fig 2B**). Among these factors, arsenic ‘concentration’ explained the most variation, followed by ‘individual’ (i.e., donor genetic background). While we randomized DO cell lines by column and MMA^III^ concentrations by row within a plate, we observed that inter-plate and inter-run effects also influence variance in measured morphological traits (**Fig 2B**). Depending on the trait, ‘individual’ explained ~0–40% of the variance with an average of 10%, suggesting that a subset of these traits (those with >20%) would provide sufficient signal for genetic mapping based on the size and architecture of our DO cell population [49,50].

While HCS produces thousands of morphological traits, many of them are highly correlated (**Fig 2C**). The correlated groups could be loosely categorized as traits describing `cell size', `γH2AX foci', `cell roundness', `intensity', and `uniformity' (**Fig 2C**). While there are a variety of dimension reduction techniques that take advantage of correlation to summarize high dimensional data, we were most interested in traits exhibiting non-linear, dose-dependent responses.

### Dose-response modeling and genetic mapping of cell morphology quantitative trait loci (cmQTL)

Dose-response models provide benchmark dose estimates of the concentrations at which an exposure to a given chemical could pose a health risk [53]. To focus on the subset of traits exhibiting dose-dependent responses, we performed dose-response modeling using the *drc* R package [54] for each cellular trait, individual, and replicate experiment (**Fig 1E**). These models provided quantitative dose-response parameters (DRPs) describing each donor individual’s cellular response including effective concentrations (EC’s), starting/maximum asymptotes, and rates of change (slopes) [55]. For example, an individual’s EC50 represents the concentration of MMA^III^ at which there is a 50% change in a given cellular feature relative to baseline. Following dose-response modeling, we applied an interplate batch correction and summarized across intraindividual replicates which resulted in 5,105 cmDRPs from 568 cellular traits (see Methods).

**Fig 3:**
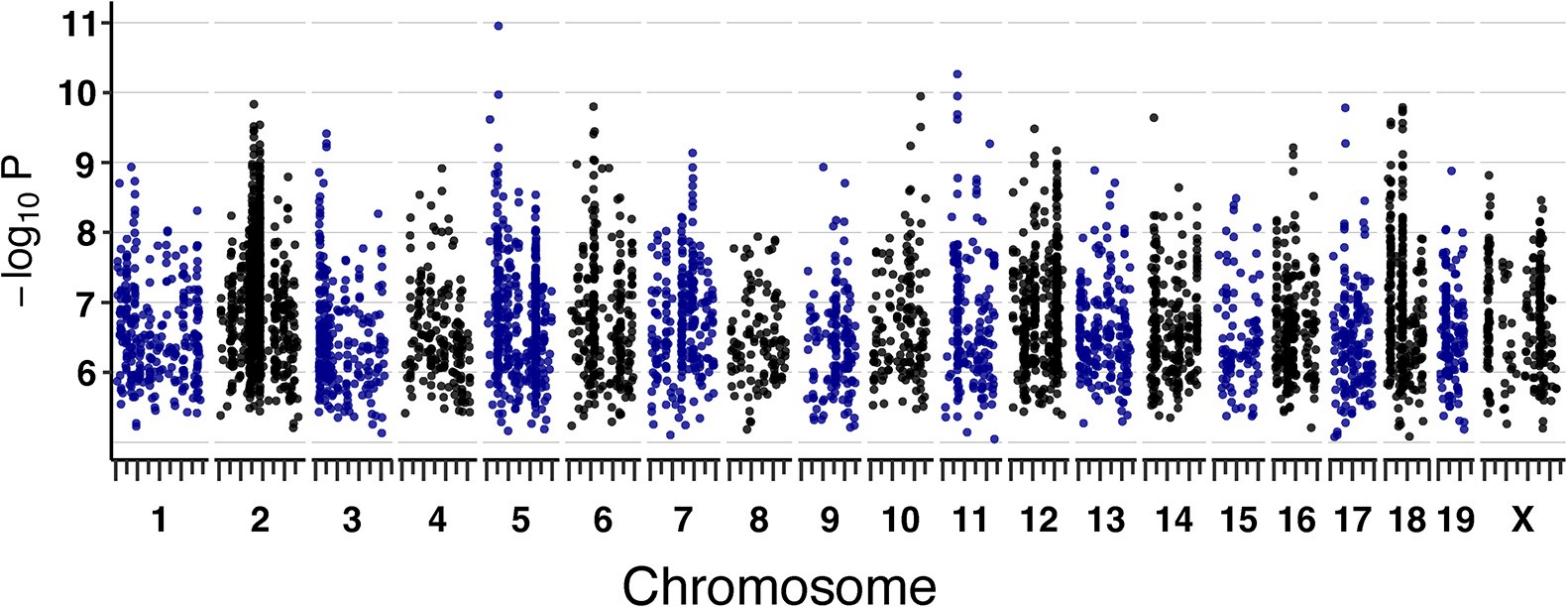
Dose-Response Modeled cmQTL in DO Fibroblasts Exposed to MMA^III^. Summary of cmQTL maximum peaks for 5100 cmDRPs. Each points represents the strength of the genetic association as a LOD score on the y-axis (-log_10_P) across the mouse genome (x-axis). On the x-axis, long tick marks represent the start of the chromosome and 50 Mbp intervals, while the short tick marks are at 25 Mbp intervals.

To reveal genetic loci that influence sensitivity to arsenic metabolite MMA^III^, we performed quantitative trait loci (QTL) mapping, treating the 5105 cmDRPs as quantitative traits (see Methods) (**Fig 1F**). To account for the data’s complicated structure and redundancy in the context of multiple testing burden, we calculated an experiment-wide, genome-wide false discovery rate (FDR) significance threshold, which resulted in one maximum peak meeting significance (FDR ≤ 10%) (**Fig 3**). Given that this work represents a proof of principle and cmDRPs are potentially noisy as modeled quantities, we also used a lenient significance threshold of LOD score > 7.5, which corresponds to ~80% power for a moderately polygenic trait in the DO [50]. Of the 5105 cmDRPs, 854 possessed suggestive genetic loci associations, with the strongest LOD score being 10.95. Chromosomes 2, 3, 6, 12, 14, 18 contained cmQTL hotspots, which contain genetic associations for numerous traits, many of which proved to be correlated (**Figs 2C** and **3**). cmQTLs with suggestive LOD scores included EC’s, slope, and maximum asymptotes, which we refer to as dose-response cmQTLs, in addition to baseline (starting asymptote) cmQTLs.

### Candidate cmQTL genes identified using differential gene expression analysis, gene set enrichment, and data integration

To nominate candidate genes and variants within cmQTL, we used several approaches. We generated bulk RNA-seq data from 16 DO fibroblast lines across 0 and 0.75 μM exposures which we used for differential expression (DE) analysis. Then we used gene set enrichment analysis (GSEA) to identify individual genes, and groups of genes, that showed differential expression in response to MMA^III^ (S3 **Table**). We interrogated published gene–arsenic interactions through the Comparative Toxicogenomics Database (CTD) [56] and we quantified the number of interaction annotations in CTD across all curated studies involving MMA^III^, MMA^V^, DMA^III^, DMA^V^, sodium arsenite, sodium arsenate, arsenic, and arsenic trioxide. For any causal variants that exert their effects through gene expression, the contributing haplotypes and direction of their effects can be correlated across eQTL and cmQTL in datasets generated from the same genetic reference population (DO). Therefore, we also correlated the cmQTL haplotype effects with previous DO eQTL from liver, heart, kidney, striatum, pancreatic islet cells, and mESCs (see Methods). Finally, local variant association mapping within each cmQTL allowed us to identify the locations of SNPs and structural variants with the highest LOD scores in each interval in relation to gene positions.

**Fig 4:**
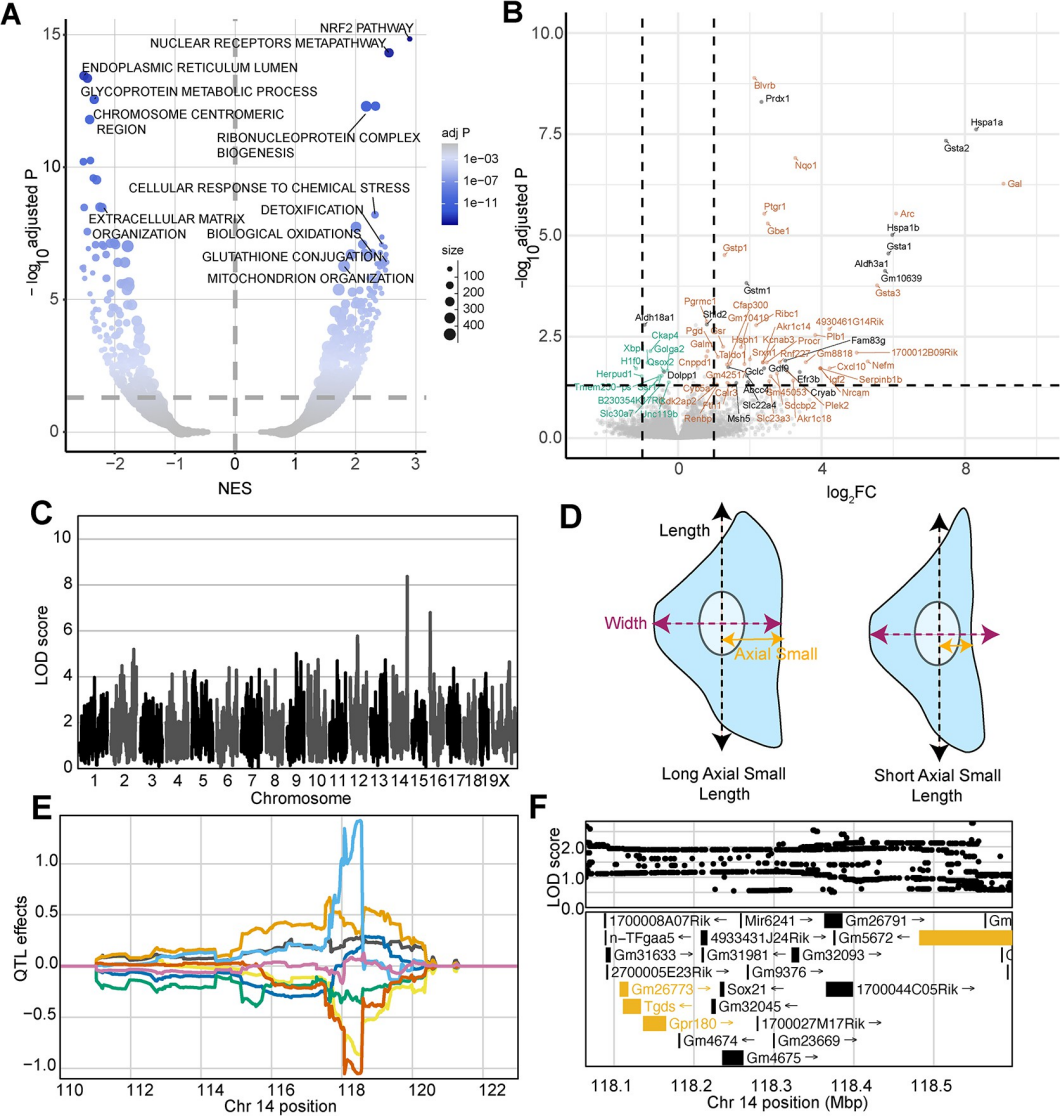
Differential Expression and cmQTL Together Support MMA^III^ Glutathione Conjugation and its Export via ABCC4. (A) Volcano plot showing the normalized effect sizes (NES) and -log_10_ Benjamini-Hochberg adjusted *p* values (adj. *P*) of the score-based gene set enrichment (GSEA) results from differential expression (DE) analysis across the 0 and 0.75 μM MMA^III^ exposed DO fibroblasts groups (n = 32, 16 individuals). Genes were considered “not expressed” if expression levels were TPM < .5 or if at least half of the points were below this cutoff. Each point represents a gene set from `GO:Component',`REACTOME', `KEGG', `WikiPathways', `GO:Tissue', `GO:Molecular Function' and `GO:Biological Process'. The size of each points represents the number of genes within the gene set and the color represents the -log_10_ adj. *P* (y-axis). Horizontal dashed line indicates the adj. *P* significance threshold (adj. *P* = 0.05). (B) Volcano plot showing the log_2_-fold change (log_2_FC) and adjusted *p* values (-log_10_ adj. *P*) for single genes. The horizontal indicates the adj. P significance threshold (adj. P = 0.05) and the vertical lines represent the ± 1 log_2_fold change for reference. Points labeled with gene names are significantly differentially expressed (adj. *P* < .05) with effect sizes > 0.75 log_2_FC or < -0.25 log_2_FC. Colors represent genes withing cmQTL confidence intervals (black), upregulated (orange) and downregulated (green) DE. (C) QTL scan for the EC05 cell MitoTracker size (‘EC05_nonborder_mitosmooth_axial_small_length_mean_per_well’) cmQTL with the maximum peak at chromosome 14:118483436 bp (GRCm38) and a LOD score of 8.36. (D) Cartoon fibroblast cells depicting the two measurements of cell length (black), width (purple), and axial small width (yellow). Fibroblast on the left has a longer axial small length compared to the fibroblast on the right. (E) Haplotype effects plot showing the eight DO founders (colors, see Methods) for the EC05 cell MitoTracker size (‘EC05_nonborder_mitosmooth_axial_small_length_mean_per_well’) cmQTL across the surrounding region on chromosome 14 (Mbp). Colors indicate founder mouse strains: A/J (yellow), C57BL/6J (gray), 129S1/SvImJ (orange), NOD/ShiLtJ (dark blue), NZO/HILtJ (light blue), CAST/EiJ (green), PWK/PhJ (red), and WSB/EiJ (purple). (F) Variant association mapping within the CI the cmQTL EC05 cell MitoTracker size (‘EC05_nonborder_mitosmooth_axial_small_length_mean_per_well’). Top panel shows the LOD scores of the known, segregating variants in the 8 DO founders (GRCm38). Bottom panel shows the gene models within the respective CI. Each point represents a variant. Colors indicate whether a gene is expressed > 0.5 TPM (gold) or < 0.5 TPM (black). The arrow indicates the direction of transcription.

At the pathway level, the most upregulated gene set in dosed samples was ‘NRF2 activation (WP2884)’, which is a well-established response to oxidative stress following arsenical exposure [57–60] (**Fig 4A**). NRF2, also known as NFE2L2, is a transcription factor that is shuttled to the nucleus following dissociation from KEAP1 in response to the generation of ROS [61–63]. In the nucleus, NFE2L2 binds antioxidant response elements (AREs) upstream of many redox homeostasis and cellular defense genes to drive their transcription in response to stress, including arsenical exposure [57,58,64–67]. These data provided multiple lines of evidence supporting *Nfe2l2* (*Nrf2*) as a candidate gene for the cmQTL hotspot that we found on chromosome 2 (**Fig 3**). Our gene expression analysis also revealed five candidate genes for other response cmQTL with LOD scores > 8 (**Fig 4B**). Three of the five genes were present within the same CI, including *Hspa1b*, *Hspa1a*, and *Msh5*, and the former two differentially expressed genes (DEGs) have over 80 previously defined interactions with arsenic metabolites in CTD. We also found that among the other DEGs 73 (89%) have not previously been associated with MMA^III^, though many have been associated with arsenic or other arsenic metabolites.

### Natural variation in cellular detoxification pathways partially explains arsenic sensitivity

The other two DEGs within dose-response cmQTL were *Cryab* and *Abcc4*, each with ≥ 19 published arsenical interactions (**Fig 4B**). SNPs in *Abcc4* have been previously associated with sensitivity to arsenic [68]. *Abcc4* encodes the protein ABCC4/MRP4, which has been shown to export glutathionylated MMA^III^ from cells [69,70]. Glutathione transferases like *Gstm1*, *Gsta1*, and *Gstp1* were also significantly upregulated in our expression dataset. These genes are members of the glutathione conjugation pathway which is a detoxification pathway that leads to glutathionylation of MMA^III^ (MMADG^III^) (**Fig 4A and 4B**) [69,71]. We found multiple cmQTLs at the *Abcc4* locus and they were all for traits related to changes in cell size (i.e., length, compactness) (**Fig 4C**). For example, one of these response cmQTL was EC5 of the change in axial small length or the dose at which 5% of the cell population exhibited measurable differences in cell size (defined by the smoothed MitoTracker labeling which captures the cytoplasmic area occupied by mitochondria) (**Fig 4D**). Variant association mapping revealed that the highest scoring SNPs in these cmQTLs were within the *Abcc4* gene, and the haplotype effects indicated that changes in cell size (‘shrinkage’) occur at lower doses in individuals with PWK haplotypes compared to those with NZO haplotypes (**Fig 4E and 4F**). Taken together, these data support a model where sensitivity to arsenic exposure in the DO population is partly regulated by natural variation in the efficiency of MMA^III^ detoxification and export.

### *Xrcc2* haplotypes modulate and predict of cellular responses

**Fig 5:**
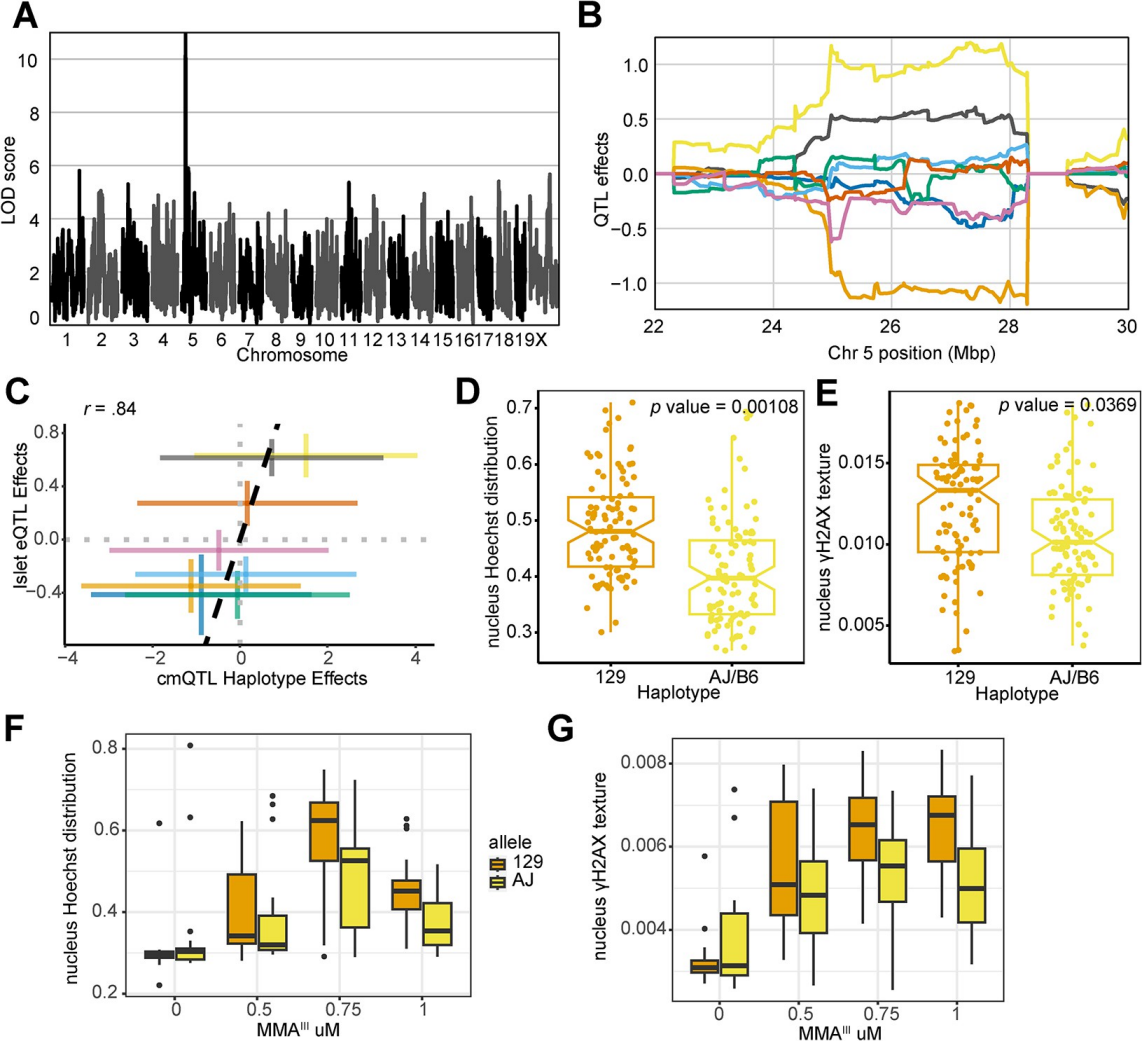
*Xrcc2* haplotype modulates chromosomal organization and DNA damage during acute MMA^III^ exposure. (A) QTL scan for the EC90 nucleus Hoechst distribution texture hole (`EC90 Hoechst Nucleus Symmetry (02) Hole Mean per Well') cmQTL with the maximum peak at chromosome 5: 27,327,254 bp (GRCm38) and a LOD score of 10.95. (B) Haplotype effects plot showing the eight DO founders (colors, see Methods) for the EC90 nucleus Hoechst distribution texture hole (`EC90 Hoechst Nucleus Symmetry (02) Hole Mean per Well') cmQTL across the surrounding region on chromosome 5 (Mbp). Colors indicate founder mouse strains: A/J (yellow), C57BL/6J (gray), 129S1/SvImJ (orange), NOD/ShiLtJ (dark blue), NZO/HILtJ (light blue), CAST/EiJ (green), PWK/PhJ (red), and WSB/EiJ (purple). (C) Pairwise correlation between the haplotype effects of *Xrcc2* expression in pancreatic islet cells at chromosome 5:27,327,254 bp (GRCm38) and the haplotype effects of the EC90 nucleus Hoechst distribution texture hole (`EC90 Hoechst Nucleus Symmetry (02) Hole Mean per Well') cellular feature (Pearson correlation coefficient (*r*) = .84). The colors are the same as in panel B. (D) Boxplot showing the nucleus Hoechst distribution texture hole cellular feature at 1 μM MMA^III^ for the top 129 (n = 24, 4 replicates each; orange) and AJ/B6 (n = 24, 4 replicates each; yellow) haplotypes and technical replicates in the DO fibroblasts. (E) Boxplot showing the γH2AX fluorescence texture bright cellular feature at 1 μM MMA^III^ for the top 129 (n = 24, 4 replicates each; orange) and AJ/B6 (n = 24, 4 replicates each; yellow) haplotypes and technical replicates in the DO fibroblasts. (F) Boxplot showing the nucleus Hoechst distribution texture hole cellular phenotype in a follow-up experiment where DO fibroblasts with 129 (n = 5; orange) and AJ (n = 5; yellow) haplotypes exposed to increasing MMA^III^ concentrations. (G) Boxplot showing the nucleus γH2AX texture bright cellular phenotype in a follow-up experiment where DO fibroblasts with 129 (n = 5) and AJ (n = 5) haplotypes exposed to increasing MMA^III^ concentrations. Colors indicate the DO founder strains (see Methods).

The cmQTL with the highest LOD score was on chromosome 5 at 27,327,254 bp (GRCm38) for the response cmQTL EC90 nucleus Hoechst distribution texture hole (EC90 Nonborder Nucleus Symmetry 02 SER Hole (Hoechst) Mean Per Well) (**Fig 5A–5D**). Hoechst nuclear fluorescence in cells with the 129 haplotype resembled apoptotic nuclei [72] and were brighter and more uniform than those found in cells with AJ/B6 haplotypes (**Figs 5B and S1A**). The highest associated SNPs for this cmQTL were located in two genes: *Actr3b* and *Xrcc2* (**S1B Fig**), however several key points suggest *Xrcc2* as the more likely candidate. First, *Xrcc2’s* paralogs, *Xrcc1* [73,74] and *Xrcc3* [75,76] have both been associated with genetic susceptibility to arsenical exposure. Second, knockdowns of *Xrcc2* were previously shown to increase both γH2AX intensity and chromosomal abnormalities [77], and *Xrcc2* is a member of the Biological Fibroblast Apoptosis (GO:0044346) and DNA Damage Repair pathways (R-MMU-5693532). Lastly, the cmQTL haplotype effects are highly correlated with an *Xrcc2* eQTL in pancreatic islets cells from the same mouse population (**Fig 5C**). Taken together, these results suggested that genetic variation at this locus may be mediating DNA damage-induced apoptosis through *Xrcc2* expression.

Because of the role in *Xrcc2* in DNA damage and apoptosis, we reasoned that γH2AX fluorescence might also be higher in cells with the more sensitive 129 haplotype compared to cells with the more resistant AJ/B6 haplotypes at the higher MMA^III^ concentrations. As expected, we observed a significant difference for fibroblasts with 129 haplotypes (n = 24, 4 replicates each) compared to individuals with AJ/B6 haplotypes (n = 24, 4 replicates each) for the nucleus Hoechst distribution texture hole phenotype at the 1.0 μM concentration based on a *t*-test accounting for the replicate structure with a random effect (*p* value = 0.0011)[78] (**Fig 5D**). Using this same technique, we also observed that ‘nucleus γH2AX texture bright’ feature (a proxy for γH2AX fluorescence) was significantly higher in the fibroblasts with the 129 haplotype compared to the AJ/B6 haplotypes (*p* value = 0.0369) (**Figs 5E and S1A**). Indeed, the ‘nucleus γH2AX texture bright’ feature was significantly higher in the fibroblasts with the 129 haplotype compared to the AJ/B6 haplotypes (**Figs 5E and S1A**). We sought to assess the reproducibility of these effects, both for the original phenotype and the increase in γH2AX. Taking advantage of our full panel of 600 cell lines, we selected an orthogonal group of lines based on their haplotype at this locus (n = 5 for each allele). Not only were we able to recreate the original nuclear symmetry difference between genetic backgrounds (**Fig 5F**), but we also observed the same γH2AX fluorescence effects that were found in the original screen (**Fig 5G**). This example shows that genetic variation in near *Xrcc2* influences sensitivity and that the haplotype effects of cmQTL have predictive value for identifying sensitive cell lines.

### Non-coding genetic variation influences TXNRD1 cell fate during induced oxidative stress

**Fig 6:**
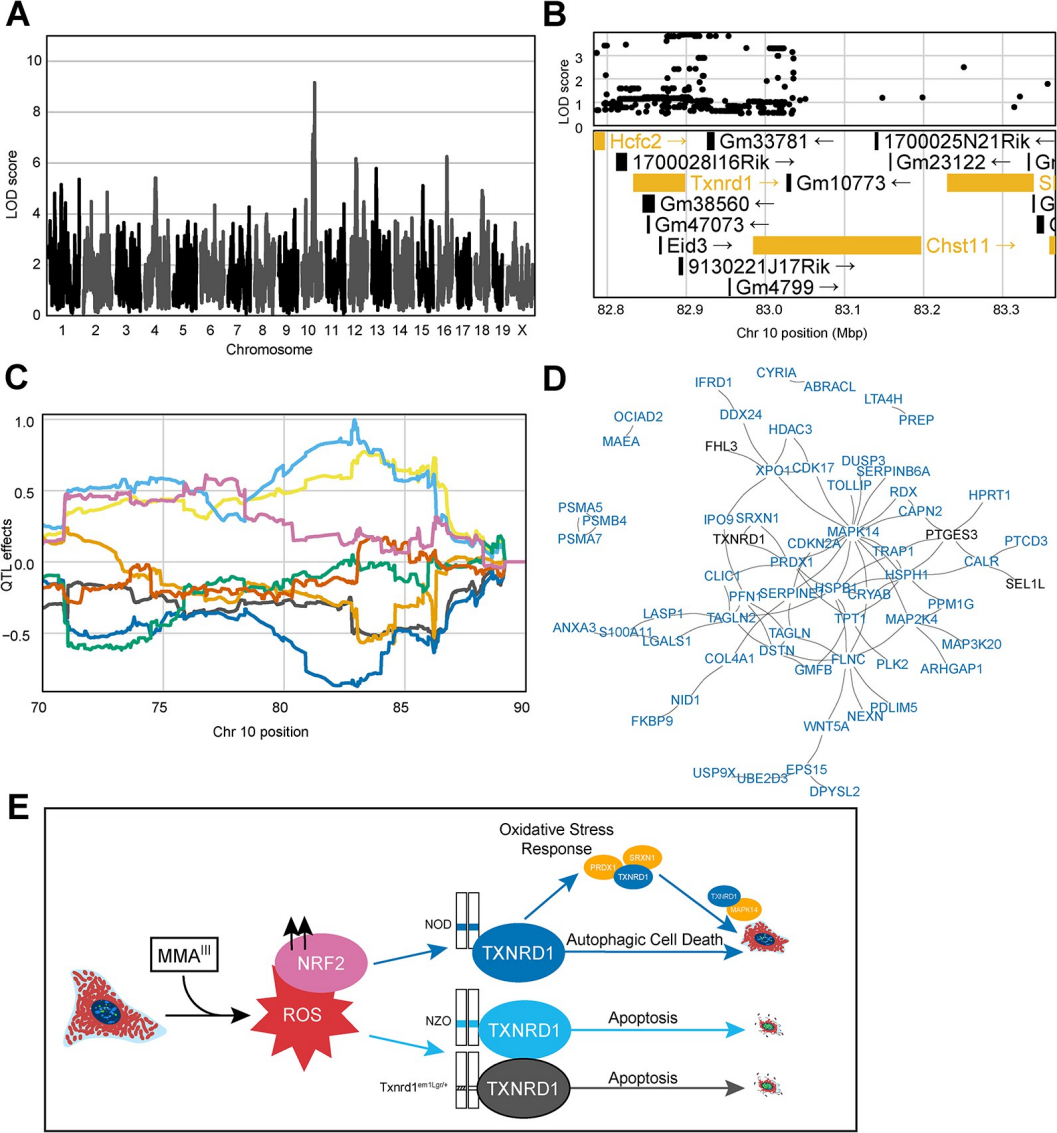
Noncoding Variation in *Txnrd1* Modulates MMA^III^-Induced Cell Death. (A) QTL scan for the `γH2AX-negative cells slope Cell Area μm^2^ mean per well' cmQTL with the maximum peak at Chromosome 10: 82,906,780 bp (GRCm38) and a LOD score of 9.16. (B) Variant association mapping within the CI the cmQTL `γH2AX-negative cells slope Cell Area μm^2^ mean per well'. Top panel shows the LOD scores of the known, segregating variants in the 8 DO founders (GRCm38). Bottom panel shows the gene models within the respective CI. Each point represents a variant. Colors indicate whether a gene is expressed > 0.5 TPM (gold) or < 0.5 TPM (black). The arrow indicates the direction of transcription. (C) Haplotype effects plot showing the eight DO founders (colors, see Methods) for the `γH2AX-negative cells slope Cell Area μm^2^ mean per well' cmQTL across the surrounding region on chromosome 10 (Mbp). (D) String-db functional enrichment network of the significantly increased protein interactors detected using immunoprecipitation mass spectrometry (IP-MS) in DO fibroblasts with NOD alleles (n = 6) at the maximum locus for the `γH2AX-negative cells slope Cell Area μm^2^ mean per well' cmQTL exposed to 0 and 0.75 μM MMA^III^ concentrations. Colors indicate whether a protein, or node, was shared with a similar experiment in DO fibroblasts with the NZO allele (n = 5). Black represents shared TXNRD1 interactors, and blue represents unique NOD-TXNRD1 interactors. (E) Mechanistic summary of allele specific *Txnrd1* responses across the NOD haplotype (blue), NZO haplotype (light blue), and heterozygous SECIS knockout model (*Txnrd1*^em1Lgr/+)^. Our data suggest DO fibroblasts with the NOD allele have a more robust oxidative stress response upon MMA^III^ exposure, ultimately succumbing to autophagic cell death represented by increased cell size at medium MMA^III^ concentrations. In comparison, DO fibroblasts with the NZO allele the *Txnrd1*^em1Lgr/+^ alleles exhibit morphology consistent with apoptosis as shown by brighter Hoechst 33342 labeling and smaller cells.

To further investigate how these data could be used for the discovery of gene–environment interactions, cmQTL mapping was performed in a subset of cells lacking accumulated DNA damage. Linear classification was performed to separate cells into γH2AX-positive and γH2AX-negative populations prior to feature extraction. To do this, we took advantage of PHENOLogic machine learning algorithms of the Harmony 4.9 software and gated the imaged cells into γH2AX-positive and γH2AX-negative populations prior to feature extraction, dose-response modeling, and mapping. We detected a cmQTL for the rate of MitoTracker area change in γH2AX-negative cells with a LOD score of 9.16 on chromosome 10 (**Fig 6A**). This locus was also detected in our original dataset with similar haplotype effects, but with a lower cmQTL LOD score of 7.71 (**S2A, S2B and S2C Fig**). Upon variant association mapping the highest LOD scoring variants were in the 3’ UTR of the *Txnrd1* gene (**Fig 6C**), a gene that is highly expressed in fibroblasts and has been previously shown to respond to arsenical exposure via changes in NRF2-mediated expression. Moreover, the reducing capacity of TXNRD1 protein is directly inhibited by MMA^III^ binding [79,80]. As a selenoprotein, the 3’ UTR of *Txnrd1* plays a crucial role in recoding a UGA stop codon into a selenocysteine amino acid which is required for function of the TXNRD1 protein as a reducing agent [81–83].

To interrogate the plausibility of *Txnrd1* as the candidate for these two cmQTL, we performed score-based GSEA using gene expression data from the bulk RNA-seq data based on their sensitive (NZO, n = 6) and resistant (NOD, n = 6) haplotypes at this locus. We found upregulation of DNA damage and replicative stress gene sets in cells with NZO haplotypes and upregulation of oxidative stress response, p38/MAPK signaling, TGF signaling, RAS signaling, lysosome, and autophagy-related pathways in cells with NOD haplotypes (**S4 Table**). Among these pathways was nanoparticle triggered autophagic cell death, which can be induced by the treatment of gold, the active component of the TXNRD1 inhibitor auranophin [84]. We did not observe signficant differential gene expression in *Txnrd1* based on the haplotype at this locus (**S5 Table**). However, there were differences in TXNRD1 protein levels in both unexposed cells (*p* value = 0.0935) and exposed cells (*p* value *=* 0.0414) between NOD and NZO haplotypes when accounting for sex and replicate using a *t*-test [78](**S2D Fig**). To assess a functional difference in TXNRD1 between these haplotypes, we performed immunoprecipitation followed by tandem mass spectrometry (IP-MS) to quantify protein-protein interactions. Following subtraction of a non-specific binding partner control, we found that compared to healthy, unexposed controls, 0.75 μM MMA^III^ exposed NOD haplotype cells (n = 6) had a larger number (106) of significant, positive interactors compared to NZO (n = 5) TXNRD1 interactors (33). We visualized these interactions using ‘string-d’ to generate a functional enrichment map of the PPIs for MMA^III^ exposed NOD and NZO TXNRD1 where we observed that there were very few genes overlapping between alleles, and that MAPK14 was a hub for NOD interactors which included proteins involved in oxidative stress (i.e., PRDX1, SRXN1) and autophagy/p38 (i.e., MAPK14, TOLLIP) (**Fig 6D and S6 Table**). Considering the gene expression and IP-MS data together, it was evident that in exposed DO fibroblasts, NOD TXNRD1 was involved in autophagy. Previous studies of *Txnrd1* deficiency have shown disruption of lysosomal-autophagy in favor of apoptotic cell death [85,86], implying an apoptotic phenotype of cells with NZO haplotypes (NZO-TXNRD1) is akin to that seen with TXNRD1 deficiency. During apoptotic cell death, cell structure and cytoskeleton are quickly degraded, but during autophagy the cytoskeleton is maintained [87–89]; providing a basis for our ability to distinguish between these two pathways and to interrogate their genetic regulation using cmQTL. Taken together, these data support a model whereby natural variation in *Txnrd1* influences the trajectory of cell death pathways following MMA^III^ exposure in the DO population (**Fig 6E**).

While we did not find coding variants unique to the NZO or NOD *Txnrd1* alleles, we found that two SNPs private to the NZO haplotype (*rs227869362* and *rs257393906*) in the 3’ UTR were adjacent to the selenocysteine insertion element (SECIS), which is essential for recoding the UGA stop codon to selenocysteine during translation [82]. We also searched publicly available data for structural variants and INDELs in the 3’ UTR but did not find any that were unique to the NZO haplotype [90]. To determine the functional consequences of variation impacting the SECIS element of *Txnrd1*, we used CRISPR/cas9 to delete the SECIS in C57BL/6J mice (*Txnrd1*^*em1Lgr*^). While heterozygous mice carrying this deletion were viable and fertile, homozygous mice could not be recovered. Since a full protein knockout of *Txnrd1* causes recessive embryonic lethality [91], we concluded that deletion of the SECIS element alone is the functional equivalent of a null allele (see Methods). We then isolated tail tip fibroblasts from heterozygous mice and found that the cell area of arsenic-exposed *Txnrd1*^*em1Lgr/+*^ fibroblasts more closely resembled fibroblasts with the NZO haplotype than their WT controls following MMA^III^ exposure (**S2E and S2F Fig**). Similarly, nuclear Hoechst 33342 labeling was brighter and more uniform in the *Txnrd1*^*em1Lgr/+*^ nuclei with increasing MMA^III^ concentration. Taken together, these data highlight the functional importance of non-coding variation in the 3’ UTR of a key selenoprotein in the context of sensitivity to arsenic induced oxidative stress. Detailed molecular and functional studies are needed to determine the impact of single nucleotide variants on Sec recoding in *Txnrd1*. However, there is at least one study demonstrating that naturally occurring and engineered single nucleotide variants in the 3’ UTR of the human selenoprotein, SEP15, influence UGA readthrough and dampen the cellular response to selenium stimulation [92].

### Natural genetic variation influences fibroblast morphology

**Fig 7:**
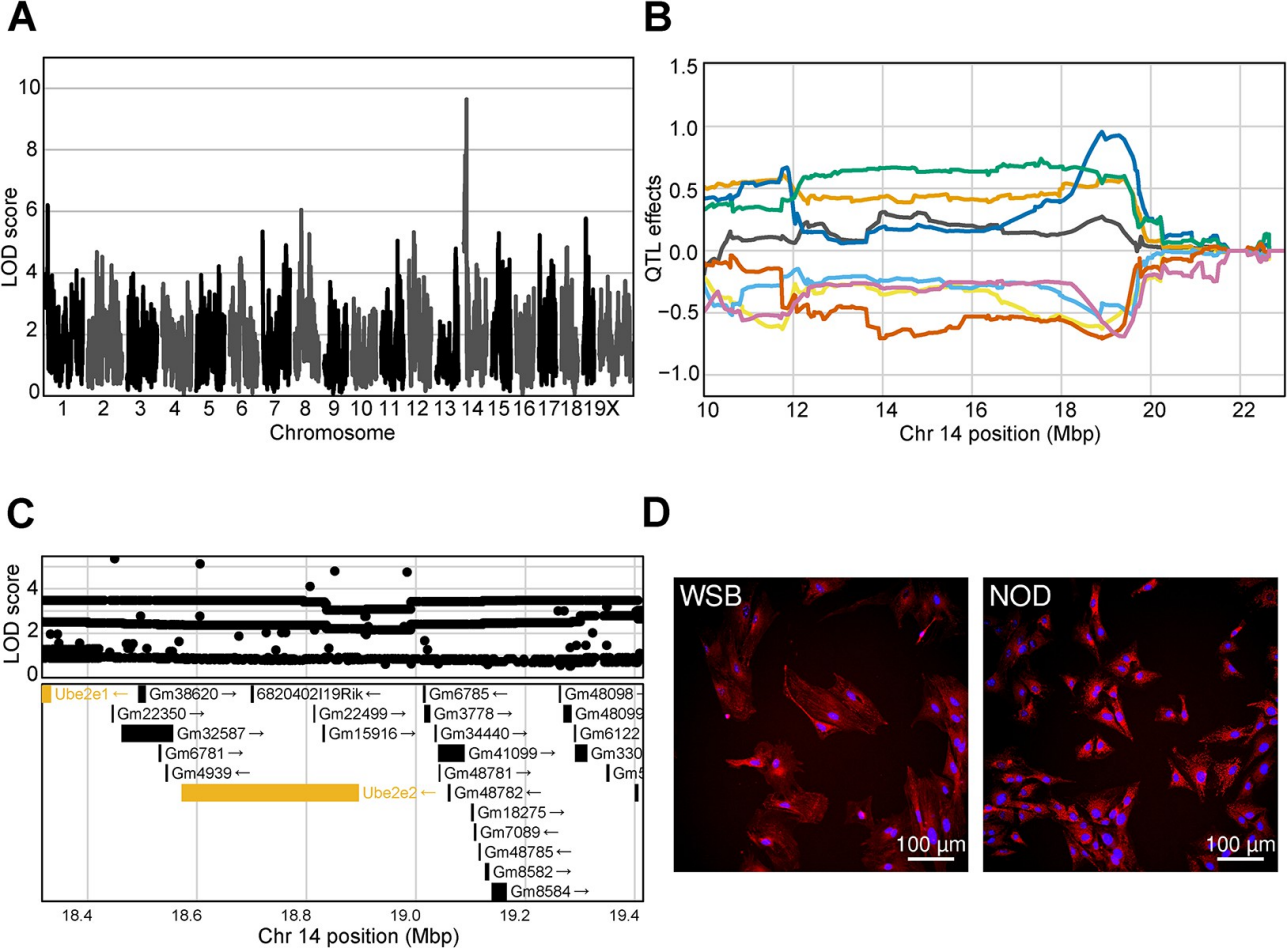
Genetic variation influences fibroblast morphology at baseline. (A) QTL scan for the baseline nucleus Hoechst texture bright (`d_nonborder_nucleus_hoechst_33342_ser_bright_1_px_mean_per_well') cmQTL with the maximum peak at chromosome 14:19401644 bp (GRCm38) and a LOD score of 9.64. (B) Haplotype effects plot showing the eight DO founders (colors, see Methods) for the baseline nucleus Hoechst texture bright (`d_nonborder_nucleus_hoechst_33342_ser_bright_1_px_mean_per_well') cmQTL across the surrounding region on chromosome 14 (Mbp). Colors indicate founder mouse strains: A/J (yellow), C57BL/6J (gray), 129S1/SvImJ (orange), NOD/ShiLtJ (dark blue), NZO/HILtJ (light blue), CAST/EiJ (green), PWK/PhJ (red), and WSB/EiJ (purple). (C) Variant association mapping within the CI the cmQTL baseline nucleus Hoechst texture bright (`d_nonborder_nucleus_hoechst_33342_ser_bright_1_px_mean_per_well'). Top panel shows the LOD scores of the known, segregating variants in the 8 DO founders (GRCm38). Bottom panel shows the gene models within the respective CI. Each point represents a variant. Colors indicate whether a gene is expressed > 0.5 TPM (gold) or < 0.5 TPM (black). The arrow indicates the direction of transcription. (D) Representative images for the two fibroblast lines showing higher Hoechst 33342 texture bright in the sample with the NOD allele at the chromosome 14 locus compared to the WSB. Nuclei are shown in blue by Hoechst 33342 labeling and mitochondria are shown in red by MitoTracker Deep Red. Scale bar indicates 100 μm.

While our primary focus was on population variation in arsenic response, we unexpectedly observed variation in fibroblast morphology in unexposed cells and our genetic analysis revealed multiple loci contributing to this baseline morphological variation (i.e. starting asymptote cmQTL). The highest scoring of these baseline cmQTL (LOD 9.64) was on proximal chromosome 14 (**Fig 7A–7B**). Several of the top LOD scoring variants were in *Ube2e2*, which was one of only three protein coding genes expressed in fibroblasts within the confidence interval (**Fig 7C**). This cmQTL is for a trait that describes the brightness of Hoechst labeling (i.e., texture feature bright 1 pixel mean per well) which is directly related to the distribution and amount of chromatin in the nucleus (**Fig 7D**) [93]. The ubiquitin conjugating enzyme E2 (UBE2E2) functions in the nucleus to post-translationally modify proteins that regulate the G1/S phase transition together with *Trim28* [94], which could explain the difference in Hoechst labeling as mitotic cells accumulate more Hoechst due to their DNA content. This example highlights the role of genetic variation in the regulation of morphology, potentially through variation in basic cellular functions (i.e. cell cycle) providing an exciting avenue for further study.

## Discussion

We created a new model for *in vitro* analysis of gene–environment interactions using cell lines from a laboratory mouse genetic reference population and performed HCS to quantify changes in cell morphology in response to acute MMA^III^ exposure. We used dose-response modeling to summarize cellular morphology changes across increasing MMA^III^ concentrations, from which we estimated individual genotype-level dose-response parameters for cmQTL mapping. We used orthogonal gene expression datasets from previous DO studies [95], pathway information, and gene–chemical interaction data from arsenicals through CTD (https://ctdbase.org) to refine our cmQTL and to identify candidate genes. A summary of these methods, and the major experimental findings from our work, can be found in **S3 Fig**. We found 70 novel gene expression signatures for MMA^III^ exposure. Among them were five significantly differentially expressed genes that were also located in our higher LOD score (> 8) cmQTL CIs, including *Abcc4*. The *Abcc4* gene is an intriguing candidate for the EC5 of the change in axial small length cmQTL based on functional activity and gene expression. Variants in the 3’ UTR of *Abcc4* can regulate its expression through impacting miRNA binding [96]. We speculate that unique variants in NZO (*rs240728821*) and PWK (*rs245333533*) may be acting similarly.

While some cmQTL result from variants that exert their effects through gene expression, others can result from variants that exert their effects post-transcriptionally, to impact protein abundance or function. Therefore, we also considered candidate genes that did not exhibit differential expression in response to MMA^III^. Taking advantage of published gene-arsenic interaction data in CTD, we identified 88 genes among our cmQTL that were previously associated susceptibility to arsenic. Only six of these genes (*Abcc4*, *Nfe2l2*, *Cbs*, *Gclc*, *Gstm1*, and *Xpc*) contained non-synonymous coding SNPs that affect the response to arsenicals in previous studies (https://ctdbase.org/). Among the remaining candidate genes there are likely to be many genes with unrecognized roles in the genetic regulation of arsenic response and these warrant further study. Our findings highlight the role of natural variation in MMA^III^ detoxification, export, and the role of variation affecting the trajectory of cell death pathways that are induced in response to ROS and DNA damage.

Our QTL analysis of machine-learning (ML) derived features (specifically slope γH2AX-negative cell area μm^2^) identified *Txnrd1*, which has multiple gene–arsenic associations annotated in CTD. Our variant association analysis revealed high LOD-scoring SNPs adjacent to the SECIS element in the 3’ UTR of *Txnrd1* that influence cell size during acute MMA^III^ exposure. We validated this finding by constructing a CRISPR-deleted SECIS element in the 3’ UTR, which recapitulated the apoptotic, shrunken cellular phenotype we observed in MMA^III^ exposed fibroblasts with NZO haplotypes. The essentiality of the SECIS element for Sec recoding, relicensing a stop codon into selenocysteine, has been previously demonstrated [83]. However, our study is the first to show that this element is also required for normal embryonic development, similar to the phenotype reported for a *Txnrd1* null allele [91]. Taken together, these results show that the deletion of the SECIS element is equivalent to complete loss of TXNRD1 function, lending support to the idea that single nucleotide variants in and around the SECIS element are likely to be detrimental to protein function. When we compared global gene expression differences between the NOD and NZO haplotypes at this locus, we found enrichment for pro-cancer signaling including RAS, TGF, and p38/MAPK signaling in the NOD haplotype compared to the NZO haplotype. This was further supported by our protein interaction data where we found increased NOD TXNRD1 affinity for MAPK14 and oxidative stress-related proteins compared to NZO, which may explain the resistance to MMA^III^-induced morphology changes. Together these data support our hypothesis that 3’ UTR variation in *Txnrd1* has functional consequences on TXNRD1 function, though direct biochemical analyses are needed to fully understand the molecular consequences of these variants.

*Xrcc2*’s involvement in the DNA damage pathway may also indicate a cancer-related outcome for the highest cmQTL related to Hoechst labeling distribution. This cmQTL region shares conserved synteny with a region significantly associated with susceptibility to arsenic-induced skin lesions in a Bangladeshi population [97]. Variant association mapping detected a non-synonymous variant in mouse *Xrcc2* (*rs3156627*) unique to the 129, sensitive haplotype that overlaps a human *XRCC2* variant (*rs2098040934*), although neither are predicted to be deleterious [98]. It is also possible that non-coding SNPs lead to differential regulation of *Xrcc2* transcript levels in AJ and 129 haplotypes, which could also explain the nuclear morphological changes following MMA^III^ exposure. If these non-coding variants are influencing gene expression, we would expect to find eQTL with matching haplotype effects. Lacking contemporaneous eQTL data from our screen, we looked to eQTL datasets generated from other DO studies and found matching haplotype effects in at least one other tissue where *Xrcc2* is expressed. This provides evidence that the variants underlying *Xrcc2* cmQTL may exert their effects through gene expression in MMA^III^ exposed fibroblasts. Integrative analyses such as these are a key advantage to working with a genetic reference population where there are a growing number of publicly available -omics and QTL datasets available to power candidate gene and pathway discovery [42,99,100].

The variants driving cmQTL could be coding or non-coding and our candidate gene / variant analyses provide several examples of this as described above. This is a key advantage of cmQTL over molecular QTL, like eQTL or protein (pQTL) which are each limited to variants that exert their effects through transcript or protein abundance, respectively. Cell morphology is one of the many cellular-level phenotypes that result from the complex interplay of gene regulatory networks, making them an inexpensive and informative phenotype to use for QTL mapping. Despite our success in identifying cmQTL and, in some case, the genes and variants affecting cellular response to MMA^III^, our study had several shortcomings that could be addressed in future work. Quantitative variation in cell morphology traits can be attributed to genetic background and dose-response, but we also found substantial unexplained residual variation in some traits. Previous studies of cell morphology in genetically diverse yeast cell populations have shown that some traits are prone to high experimental variability, especially for features that have high cell-to-cell variability [6]. Since our features are whole-well summaries, cell-to-cell variability is likely a major contributor to our observed residual variation. We also found that the γH2AX features had high residual variation that is likely due to the indirect immunolabeling method used for γH2AX detection, which is a multistep staining method that relies on two antibodies and is known to have more experimental variability than direct labeling with a fluorophore-conjugated primary antibody. Other studies mapping cmQTL were limited by lack of genetic diversity [9,48]. While our study successfully addressed these issues [50,95,101], we found that it was underpowered for detecting QTL effects explaining less than 20% of trait variation and future studies would benefit from technological advances that reduce residual variation.

To induce cell morphology changes that were sufficiently extreme to fit the asymptotes of a sigmoidal dose-response curve, we used concentrations of MMA^III^ that are unlikely to be encountered through environmental or occupational exposures. Other studies have shown that cell morphology was impacted following lower concentration and longer exposures of arsenic [102], so an important future direction is to explore the differences between acute and chronic exposures. Another statistical challenge arose because existing dose-response modeling software does not allow for direct inclusion of batch effects, and thus, our study design required a post-hoc adjustment of estimated dose-response parameters. This problem may be addressed in future releases of R/drc [54]. We further acknowledge that dose-response modeling results can vary based on the choice of software, the form of the model, and as we observed, the genetic background of the samples [103]. Despite these challenges, we identified hundreds of loci where natural genetic variation in the DO founder strains influences the fibroblast responses to MMA^III^ and baseline fibroblast morphology.

Our *in vitro* system used primary fibroblasts, which are a highly abundant cell type that contribute to a diverse range of cellular and tissue functions [104,105]. However, the genetic effects in fibroblasts may not always recapitulate the same molecular mechanisms of sensitivity and resistance as those found in other specialized cell types. Primary fibroblasts are also a limited resource because they will undergo senescence, and they are more difficult to genetically manipulate than transformed cell lines or pluripotent cells. For these reasons, we have generated induced pluripotent stem cell (iPSCs; n = 284) from this panel for future work. iPSCs also enable differentiation into other cell types, 3-dimensional cell models, organoids, or scaffolded arrays, which can be screened across a variety of environmental conditions, including other toxicants, drugs, or other culture conditions.

In conclusion, our study demonstrates that dynamic changes in cell morphology occurring in response to acute exposure to MMA^III^ across a population of genetically diverse cells exhibit predictable dose-response relationships. These relationships display inter-individual variation and genetic mapping of individual-level dose-response parameters can identify genetic variation that regulates the molecular initiating events that occur during an acute exposure. Our findings indicate that these genetic loci and their associated effects have predictive value for identifying sensitive and resilient individuals *in vitro*. While further work is needed to explore the applicability of these predictions to *in vivo* responses, cell lines derived from mouse genetic reference populations present an exciting opportunity for iterative *in vitro* screening and precise *in vivo* testing of genetically encoded susceptibility and resistance to the effects of toxic exposure.

## Materials and methods

### Ethics statement

All procedures involving laboratory mice were approved by The Institutional Animal Care and Use Committee of The Jackson Laboratory (under Animal Use Summary #20030).

### Fibroblast derivation

We modified a previously described protocol for deriving fibroblasts by biopsying 2–3 mm tail tips from adult male and female DO (RRID:IMSR_JAX:009376) mice, aged approximately 4–6 weeks, using a procedure approved by The Jackson Laboratory’s Institutional Animal Care and Use Committee [106]. Samples were initially collected into Advanced RPMI 1640 cell culture media (Gibco) supplemented with 1.0% penicillin/streptomycin (P/S) (Gibco), 1.0% GlutaMAX-I (GlutaMAX)(Gibco), 1.0% MEM Non-Essential Amino Acids (NEAAs)(Gibco), and 0.0005% 2-mercaptoethanol (BME)(Gibco). Tail tissue was minced using razor blades and digested in media containing collagenase D (Gibco) at a concentration of 2.5 mg/ml on an orbital shaker at 37°C. The digested samples were further fragmented using micropipettes ranging from p1000 to p200 and dissociated in RPMI 1640 media containing 1.0% P/S, 1% Glutamax, 1.0% non-essential amino acids, 0.0005% BME, and 10% fetal bovine serum (FBS)(Gibco), hereinafter referred to as ‘fibroblast media’, for approximately 3–5 days (passage number 0; P0). All passaging was done using a phosphate-buffered saline pH 7.2 (1X, PBS) wash and 0.05% Trypsin-EDTA (Trypsin) (Gibco). Individual DO fibroblast samples were expanded to P5 with reserves frozen at approximate densities of 3.5 x 10^5^ cells/ml at passage numbers P2, P3, and P5 in freezing media containing RPMI 1640 with 10% dimethyl sulfoxide (DMSO) and 10% FBS. All DO fibroblast samples were transferred to liquid nitrogen holding tanks for long-term storage after controlled freeze (-1°C/min) 24–48 hours at -80°C.

### Genotyping

DNA was collected from the spleen tissue of each DO mouse and genotyped using the Giga Mouse Universal Genotyping Array (GigaMUGA; [107]). The haplotypes were reconstructed using a hidden Markov model to estimate genotype probabilities at each locus for the population, as described previously [108].

### Exposure of Fibroblasts to MMA^III^ and sample preparation

Frozen aliquots of P5 fibroblast lines were thawed and grown in fibroblast media in 60-mm tissue culture-treated plates for 48 hours. Trypsinized cells were resuspended and viable cell density was estimated using Trypan Blue and a Nexcelom Cellometer Auto T4 Plus Cell Counter. 100 μl of each fibroblast line was seeded into four columns (four technical replicates) distributed across two CellCarrier Ultra 96-well black, clear bottom, tissue culture treated microplates using the Integra Assist Plus at a density of ~2500 viable cells/well after randomization across columns. After 24 hours, the fibroblast media was replaced with 100 μL of fibroblast media containing monomethylarsonous acid (MMA^III^; Toronto Research Chemicals) at the concentrations 0 μM, 0.01 μM, 0.1 μM, 0.75 μM, 1.0 μM, 1.25 μM, 2.0 μM, and 5.0 μM across plates and rows.

After the cell lines were exposed to MMA^III^ for 24 hours, the media was replaced with media containing MitoTracker Deep Red (200 nM; Invitrogen), and the cells were incubated at 37°C for 20 minutes in 96-well plates. The cells were then fixed in ice-cold 100% methanol on ice for 10 minutes. After the cells were washed three times with PBS, they were bathed in a 1.0% bovine serum albumin (Fraction V) (BSA), 0.1% Tween solution overnight at 4°C on a shaker. Blocking solution was then replaced with anti-γH2AX (phosphorylated S139) antibody (Abcam, ab11174, 1:2000) in the blocking solution and incubated at room temperature for 2 hours on a shaker. After washing the cells three times with PBS, Alexafluor 488 donkey anti-rabbit secondary antibody (1:2000; Abcam) was added for 1 hour at RT on a shaker in blocking solution. Next, Hoechst 33342 (1:8000; Abcam) was added to the cells and incubated for 10 minutes at RT on a shaker. The plates were subsequently washed, and 100 μL of PBS was left in each well for storage at 4°C and imaging.

### Automated image acquisition

96-well plates were imaged using an Operetta CLS for the MMA^III^ screen and *Xrcc2* follow-up experiments equipped with a 20x/1.0 water immersion objective and binning 2. A single z-plane was acquired from 25 contiguous fields per well. Exposure times, focal heights, and excitation power settings for the Operetta CLS screen were: Hoechst 33342 (time: 100 ms, power: 100, height: -5), Alexa 488 (time: 200 ms, power: 100, height: -5), MitoTracker Deep Red (time: 500 ms, power: 100, height: -5). Exposure times, focal heights, and excitation power settings for the *Xrcc2* follow-up experiments were: Hoechst 33342 (time: 300 ms, power: 100, height: -6), Alexa 488 (time: 80 ms, power: 100, height: -6), MitoTracker Deep Red (time: 200 ms, power: 100, height: -6). Lastly, *Txnrd1*^*em1Lgr/+*^ and control fibroblast samples were imaged using the Opera Phenix High-Content Imaging System with a 20x/1.0 water immersion objective, binning 2, and a single z-plane with 25 contiguous fields per well. Exposure times, focal heights, and excitation power settings were as follows: Hoechst 33342 (time: 100 ms, power: 80, height: -10) and MitoTracker Deep Red (time: 40 ms, power: 50, height: -10).

### Image analysis / cellular segmentation

Flatfield corrected images were analyzed and processed using Harmony 4.9 software with PhenoLOGIC (PerkinElmer). Gaussian smoothed images were used for image segmentation, with a focus on two main regions of interest (ROIs), including using Hoechst 33342 to define the nucleus and MitoTracker Deep Red to define the cytoplasm surrounding each nuclear ROI. Fluorescence patterning (i.e., texture) and intensity were measured in the nuclear and cytoplasmic regions using the Hoechst 33342, γH2AX/Alexa-488, and MitoTracker Deep Red/MitoTracker Deep Red Gaussian smoothed channels. Features including nuclear area, Hoechst 33342 intensity, and nucleus edge texture were extracted and represented as mean +/- SD per well. Additionally, a spot analysis was performed using the γH2AX/Alexa-488 labeling in the nucleus. The second image analysis approach used the PhenoLOGIC machine learning (PerkinElmer) algorithms in the Harmony 4.9 software to define sub-populations of cells based on γH2AX/Alexa-488 (γH2AX-positive and γH2AX-negative) and MitoTracker Deep Red (stressed and unstressed) fluorescence prior to feature extraction to generate features including ‘MitoTracker Cell Area in γH2AX negative cells’. For more information about the specific Harmony HCS features, an Image Analysis Guide may be requested from PerkinElmer. More background information for image-based profiling and HCS features can be found through the CellProfiler documentation [109].

### Feature variance and relatedness

Principal components analysis was performed on the cellular features across all concentrations, individuals, and plates using the `pca' function from the R *pcaMethods* with the option `scale = “uv”'. Variance component analysis was performed using the ‘lmer’ function from the R package *lme4*. The sources of variation included in the model were sex, DO generation (‘generation’), DO donor (‘individual’), 96-well plate (‘plate’), and run (See **Eq 1**). Variance components were extracted from the model using the function ‘VarCorr’ for each of the random effects (generation, sex, individual, and plate). Residual variance was extracted as the sigma from the model summaries. Ratios of the variance components were determined by dividing each variance component by the sum of all the variance components and the residual variance.


$$y_{i}=(1|\mathrm{sex})+(1|run)+(1|\mathrm{generation})+(1|\mathrm{mouse})+(1|\mathrm{plate})+\varepsilon_{i}$$ Eq 1

Lastly, the pairwise correlation structure of these data was calculated using the `cor' function in the *WGCNA* R package with the option `use = "pairwise.complete.obs"'. The heatmap was created using the *ComplexHeatmap* R package, and the dendrogram added using the `column_split' and `row_split' options each set to 5. We added terms to the heatmap clusters based on a qualitative examination of the clustered trait names.

### Cellular feature dose-response modeling

The *drc* R package [54] was used to perform dose-response modeling for the 673 cellular features. For each of the 226 individuals and cellular features, four dose-response models for each technical replicate column were fit to the four-parameter log-logistic dose-response model (see Eq 2) using the ‘drm’ function with the ‘fct’ set to ‘LL.4’ with log-normalized cellular features using the ‘bcVal = 0’ option. Based on Eq 2 [54] where *x* represents concentration, *b* represents the slope, *d* represents the upper asymptote, *c* represents the lower asymptote, and *e* represents the EC50, these model parameters were extracted from the summary of the model fits. Additionally, the ‘ED’ function was used to estimate the EC5, EC10, EC25, EC75, and EC90 for each model fit ‘relative’ to the asymptotes. Four replicates for each model fit parameter summary were estimated for each DO individual and cellular feature.


$$f(x,(b,c,d,e))=c+\frac{d-c}{1+exp(b(\log\left(x\right)-\log\left(e\right)))}$$ Eq 2

### LMM / BLUP estimation

The dose-response parameter replicates for each DO individual’s concentration response parameters were summarized using Eq 3 using a linear mixed effects model (LMM) to adjust for batch effects using LMM. The LMM was fit using the ‘lmer’ function from the R package *lme4*. Each cellular feature was modeled where *y_i_* is the dose-response parameter estimate for a given cellular feature and where each DO individual (*i*) was modeled with varying intercepts through random effects for mouse/individual and 96-well plate. The random error term ε_*i*_ is assumed to ε_*i*_ ~ N(0, σ^2^), and σ^2^ is the error variance. Data without the effect of plate were extracted as the best linear unbiased predictors (BLUPs) of the random effect for DO individual and used for QTL mapping analysis.


$$y_{i}=1+(1|\mathrm{mouse})+(1|\mathrm{plate})+\varepsilon_{i}$$ Eq 3

### Cellular feature QTL mapping

All data were converted to the normal quantiles calculated from the ranked data, i.e., the rank-based inverse normal transformation (rankZ) to force a Gaussian distribution for mapping. QTL mapping was performed using the *qtl2* R package. Briefly, a genetic relationship matrix (i.e., kinship matrix) was calculated from the genotype probabilities using the ‘calc_kinship’ function with the ‘leave one chromosome out’ (loco) option for genetic mapping and the “overall” option for heritability (*h*^*2*^) estimation. Sex and DO generation were included as covariates that were assigned binary values in the LMM for QTL mapping.

For QTL mapping, whole genome scans were performed by first testing individual loci spanning the genome for association with each cellular feature (using qtl2’s ‘scan1’ function). The haplotype effects were then estimated at detected QTL as BLUPs (using the ‘scan1blups’ function) to identify the parental haplotypes driving each QTL and their respective directionality. SNP-association mapping was performed using the ‘scan1snps’ function and the known variants across the eight founder strains of the DO (https://doi.org/10.6084/m9.figshare.5280229.v3). Genome-wide FDR = 0.10 was calculated using the permutations (n = 100,000) for the ‘EC50 number of nuclei’ trait as simulated permutations for all 5105 cmDRPs mapped.

### RNA-seq of fibroblast samples

32 fibroblast cell lines, including those with NOD (n = 6), NZO (n = 6), and NOD/NZO heterozygous (n = 4) haplotypes at Chr10:82.89 Mbp (GRCm38) were thawed into 60-mm cell culture-treated plates and grown to confluency (≥ 0.8 x 10^6^ cells/ml) in fibroblast media. Each cell line was then passaged equally into two 60-mm cell culture dishes and grown to 75% confluency, upon which one 60-mm dish received 0.75 μM MMA^III^ containing fibroblast media and one 60-mm dish received standard fibroblast media. Following 24-hr exposure, both treated and untreated samples were independently collected as cell pellets and snap frozen on dry ice for 15 minutes. Samples were stored at -80°C prior to RNA isolation. RNA was extracted using a NucleoMag RNA Kit (Macherey Nagel) and purified with a KingFisher Flex system (ThermoFisher). Library preparation was enriched for polyA containing mRNA using the KAPA mRNA HyperPrep Kit (Rocher Sequencing and Life Science). Paired-end sequencing was performed with a read-length of 150 bp on an Illumina NovaSeq 6000. Genes were tagged as “not expressed” and filtered out if the the median TPM was < .05 for half or more of the samples and the remaining expressed genes were highlighted in “gold” in the variant association mapping plots.

### Transcriptomic profiling

Genotypes for each sample were reconstructed using the genotype by RNA-seq pipeline (GBRS) and aligned to the 8 founder allele-specific genome using GBRS RNA-seq pipeline to quantify read counts for each gene [110] (available through GitHub at TheJacksonLaboratory/gbrs_nextflow). These expected counts were the input for differential expression between the 0 and 0.75 μM exposures using the R package *DEseq2* [111]. The *fgsea* R package was then used to perform a score-based gene set enrichment analysis [112]. The input for GSEA was the exposure-based log_2_ fold-change for each gene normalized by its standard error. Gene Ontology (GO), REACTOME, WikiPathways, and Biocarta genesets for *Mus musculus* were obtained via the R package *msigdb* [113]. Additionally, the R package *ClusterProfiler* was used to assess enrichment of the significant differentially expressed gene set based on the outlying alleles for the cmQTL on chromosome 10 (GRCm38) [114].

### CTD database mining

The Comparative Toxicogenomics database (CTD) was used to identify gene–arsenic interactions previously defined for candidate genes within cmQTL CIs. The gene–arsenic interactions were downloaded for these arsenicals: monomethylarsonic acid (MMA^V^), monomethylarsonous acid (MMA^III^), dimethylarsinic acid (DMA^V^), dimethylarsinous acid (DMA^III^), arsenic trioxide (ATO), sodium arsenite, sodium arsenate, and elemental arsenic (As). NCBI gene ID’s were then merged to Ensembl IDs and their mouse orthologs obtained through Ensembl’s BioMart tool [115]. The number of `Interactions' were aggregated for each gene across the arsenicals to get an `Interaction Count' for the genes within cmQTL CIs.

### Comparing haplotype effects to eQTL across tissues

To determine whether cmQTLs colocalized with eQTLs from other DO tissues, Pearson correlation coefficients and Spearman correlation coefficients were calculated between the haplotype effects of eQTLs across liver, kidney, heart, bone, mESCs, striatum, and pancreatic islet cells which are all publicly available, published datasets that can be accessed through DODB (https://dodb.jax.org/) and all genes within the cmQTL CIs (< 8 Mb wide) that were shown to be expressed in fibroblasts [95]. cmQTL and eQTL haplotype effects were calculated using the coefficients from the ‘fit1’ function in the qtl2 R package.

### TXNRD1 relative abundance

DO fibroblasts were selected based on their genotypes at the *Txnrd1* locus, representing 6 NOD, 5 NZO, and 4 NOD/NZO haplotypes balanced for both male and female lines. Each line was split into two 60 mm dishes where one 60 mm plate received 0 μM MMA^III^ containing media (unexposed) while the other contained 0.75 μM MMA^III^ containing media. After 24 hours, cell pellets split into two vials and snap frozen on dry ice for further processing and liquid chromatography tandem MS (LC-MS/MS) analyses. Protein pellets were resuspended in 150 uL of 50 mM HEPES, pH 7.4, and lysed by passing through a syringe with 28 gauge needle (10 passes), vortexing for 30 seconds, and waterbath sonicating for 5 minutes (30 seconds on, 30 seconds off). Lysates were then clarified via centrifugation at 21,000 x g for 10 minutes at 4°C. Clarified lysates were quantified using a microBCA assay and 20 μg samples were diluted to 50 uL for digestion in 50 mM HEPES, pH 8.2. Samples were then reduced with 10 mM DTT at 37°C for 30 minutes, alkylated with 15 mM IAA at room temperature in the dark for 20 minutes, and trypsin digested overnight at 37°C (trypsin:protein ratio of 1:50). Samples were then cleaned-up using Millipore P10 zip-tips, dried in a vacuum centrifuge, reconstituted in 20 μL of 98% water/2% ACN with 0.1% formic acid, and transferred to mass spec vials. Each sample was analyzed using Thermo Eclipse Tribrid Orbitrap Mass Spectrometer coupled to a nano-flow UltiMate 3000 chromatography system on a Thermo 50 cm EasySpray C18 column as described previously with the exception that the gradient was scaled down to a 90 minute gradient [116]. TXNRD1 abundance was determined based on the target peptide: IEQIEAGTPGR. Raw peak data was processed using Skyline (version 22.2.1.278) and further analyzed in R. All mass spectrometry analysis was performed in the in The Jackson Laboraory (JAX) Mass Spectrometry and Protein Chemistry Service.

### Immunoprecipitation mass spectrometry (IP-MS)

Immunoprecipitation mass spectrometry (IP-MS) was performed using a rabbit antibody derived against the mouse TXNRD1 protein gifted from Dr. Edward Schmidt (Montana State University) to determine TXNRD1 binding partners using the samples and instrumentation described in the ‘TXNRD1 Relative Abundance’ section. M-280 Sheep Anti-Rabbit IgG Dynabeads (Invitrogen, 11203D) were prepared and coupled to the rabbit anti-mouse TXNRD1 antibody according to manufacturer protocol; additional IgG control beads with no TXNRD1 were also prepared as a non-specific binding partner control for the beads. A ratio of 5 ug of antibody to 5 x 10^7^ beads was used. All Dynabeads were then blocked with 5 mg/mL BSA overnight at 4°C during the antibody coupling step. Coupled and control IgG Dynabeads were then bound to 250 μg of protein lysate at room temperature with rotation for one hour. Heterozygous samples were pooled and used as IgG subtractive controls to assess non-specific binding for the beads. All bound bead fractions were clarified with a magnet, then washed three times with Wash Buffer A (10 mM HEPES at pH7.4, 10 mM KCl, 50 mM NaCl, 1 mM MgCl2, NP-40 (0.05% w/v)), followed by two washes with Wash Buffer B (10 mM HEPES at pH7.4, 10 mM KCl, NP-40 (0.05% w/v)). Washed beads were then digested on-bead as described for the relative abundance section above with the exception of 500 ng of trypsin being used. Samples were then purified using a Millipore P10 Zip-tip and prepped for tandem mass spectrometry analysis, both as described above in the relative abundance section. Raw data was analyzed using the Thermo Proteomic Discoverer software as described previously in the JAX Mass Spectrometry and Protein Chemistry Service using standard operating protocols [116].

### PPI and functional enrichment

The *string_db* R package was used to assess the functional enrichment of proteins binding TXNRD1 to generate protein-protein interaction (PPI) networks for the allele-specific IP-MS results [117]. A score threshold of ‘400’ was used to identify functional interactions between TXNRD1-interacting proteins (nodes) across NOD and NZO haplotypes at the Chromosome 10 locus, which were indicated as edges in the *igraph* R package visualization. The PPI was colored based on shared (black) and unique (blue) proteins across alleles.

### Deletion of *Txnrd1* SECIS element

To delete a 200-bp region containing the 75-bp SECIS translational regulatory element of *Txnrd1* (MGI:1354175, NCBI Gene: 50493, ENSMUSG00000020250) as well as the flanking regions where 3’ UTR variants are found in NZO haplotypes, C57BL/6J (The Jackson Laboratory stock #000664, RRID:IMSR_JAX:000664RRID:JAX000664) embryos were engineered using CRISPR/Cas9. The murine SECIS element of *Txnrd1* is located at 1967–2042 bp in the *Txnrd1* mRNA NM_001042513.1. Two sets of gRNAs were used (gRNA up 1:GGAGGCTGCAGCATCGCACT, gRNA down 1: GGGTTAATGATACTAGAGAT, gRNA up 2: GAGGCTGCAGCATCGCACTG, gRNA down 2: GGTTAATGATACTAGAGATA) with no repair template. Off-target effects were assessed using the Benchling algorithm (https://benchling.org) and for all gRNAs, potential off-target sites were scored <2.0. Two F0 founders (male 5007 and female 5016) carrying the expected 220-bp deletion at chr10:82,896,230–82,896,450 (GRCm38) were identified by PCR. PCR genotyping primers were designed to amplify a 565-bp WT product and a 365-bp deletion product (SECIS_500_FWD 5’ CCTTCCTCTTT CTGCAGATATT 3’, SECIS_500_REV 5’ ACC CAC TTCCACACAGTAAAG 3’). Male founder 5007 was backcrossed to C57BL/6J females and PCR genotyping (primers) was used to identify N1 heterozygous offspring. After two more backcrosses N3 animals were intercrossed to generate N3F1 and N3F2 animals for phenotyping and tail tip fibroblast biopsy. The heterozygous crosses resulted in 320 animals, 211 animals were heterozygous (66%), 109 were wildtype (34%) and 0 were homozygous for the deletion allele. This 2:1 Mendelian ratio (het:WT) was consistent with recessive embryonic lethality of the deletion allele. Targeted nanopore sequencing (Oxford Nanopore Technologies) of the flanking genomic region was used to confirm the expected SECIS deletion and to confirm the lack of closely linked off-target mutations the coding and intronic regions of the *Txnrd1* gene. The resulting strain C57BL/6J-*Txnrd1*^*em1Lgr*^/Lgr was assigned The Jackson Laboratory stock #37668. All experiments using mice were approved by The Jackson Laboratory’s Institutional Animal Care and Use Committees.

## Supporting information

S1 Fig Genetic variation near *Xrcc2* associated with nuclear changes following MMA^III^ exposure. (A) Representative images for the two fibroblast lines at a 1.0 μM MMA^III^ concentration with nuclei labeled by Hoechst 33342 (blue) and γH2AX (Alexa-488 secondary; green) for primary fibroblasts with a 129 allele (orange; n = 3) versus an AJ/B6 allele (yellow; n = 3) at the maximum position for the EC90 nucleus Hoechst distribution texture hole (`EC90 Hoechst Nucleus Symmetry (02) Hole Mean per Well') cmQTL. (B) Variant association mapping within the CI the cmQTL EC90 nucleus Hoechst distribution texture hole (`EC90 Hoechst Nucleus Symmetry (02) Hole Mean per Well'). Top panel shows the LOD scores of the known, segregating variants in the 8 DO founders (GRCm38). Bottom panel shows the gene models within the respective CI. Each point represents a variant. Colors indicate whether a gene is expressed > 0.5 TPM (gold) or < 0.5 TPM (black).
(TIF)

S2 Fig Heterozygous SECIS-Knockout in *Txnrd1* Recapitulates Cell Area Phenotype. (A) QTL scan for the EC90 cell MitoTracker distribution (EC90_nonborder_mitosmooth_symmetry) cmQTL with the maximum peak at chromosome 10:82,967,807 bp (GRCm38) and a LOD score of 7.64. (B) Haplotype effects plot showing the eight DO founders (colors, see Methods) for the EC90 cell MitoTracker distribution (EC90_nonborder_mitosmooth_symmetry) cmQTL across the surrounding region on chromosome 10 (Mbp). (C) Variant association mapping within the CI the cmQTL `γH2AX-negative cells slope Cell Area μm^2^ mean per well'. Top panel shows the LOD scores of the known, segregating variants in the 8 DO founders (GRCm38). Bottom panel shows the gene models within the respective CI. Each point represents a variant. Colors indicate whether a gene is expressed > 0.5 TPM (gold) or < 0.5 TPM (black). The arrow indicates the direction of transcription. (D) Relative abundance of TXNRD1 compared between DO fibroblast lines with NOD (n = 6), NZO (n = 5), and NOD/NZO (n = 4) alleles at the chromosome 10 locus. (E) MitoTracker Deep Red Cell Area across increasing MMA^III^ concentration for *Txnrd1*^em1Lgr/+^ (n = 3) compared to B6 control (n = 3) primary fibroblasts. Colors indicate wild-type (black) compared *to Txnrd1*^em1Lgr/+^ (gray) primary fibroblast lines. (F) `Hoechst 33342 intensity' across increasing MMA^III^ concentration for *Txnrd1*^em1Lgr/+^ (n = 3) compared to B6 control (n = 3) primary fibroblasts. Colors indicate wild-type (black) compared to *Txnrd1*^em1Lgr/+^ (gray) primary fibroblast lines.
(TIF)

S3 Fig Summary of methods and findings. Primary fibroblasts were derived from Diversity Outbred (DO) mice and were exposed to 8 increasing concentrations of MMA^III^. They were labeled with Hoechst 33342, MitoTracker Deep Red, and γH2AX/488, imaged using the Operetta (PerkinElmer) at 20X, and images were analyzed using Harmony 4.9 to yield 673 HCS cellular features. These cellular features were then fit to a log-logistic dose-response model where parameters were extracted including the starting asymptote, slope, EC5, EC10, EC25, EC50, EC75, EC90, and maximum asymptote to yield. Following linear mixed modeling summarization of replicates and inter-plate batch correction, 5105 cellular traits remained for cmQTL mapping. Whole genome scans were performed across all 5105 cellular features to identify cellular morphology quantitative trait loci (QTL) influencing fibroblast sensitivity to MMA^III^ where 1 cmQTL reached significance (FDR ≤ .1) and 854 cmQTLs had suggestive LOD scores > 7.5. Differential expression, gene set enrichment, previous gene-arsenical interactions curated from the Comparative Toxicogenomics Database (CTD), and correlated haplotype effects between cmQTL and DO eQTL data across many tissues were used to nominate candidate genes and variants.
(TIF)

S1 Table Dose-response cmQTL maximum peaks. (CSV)

S2 Table Differential expression analysis in fibroblasts exposed to MMA^III^. (CSV)

S3 Table Gene-set enrichment analysis in fibroblasts exposed to MMA^III^. (CSV)

S4 Table *Txnrd1* haplotype-specific gene-set enrichment analysis. (CSV)

S5 Table *Txnrd1* haplotype-specific differential expression analysis. (CSV)

S6 Table *Txnrd1* haplotype-specific immunoprecipitation mass spectrometry (IP-MS) following 0.75 μM MMA^III^ exposure. (XLSX)

S7 Table Summary of dose-response cmQTL candidate gene support. (CSV)
