# Supplementary figures and images for: Unraveling the genetics of arsenic toxicity with cellular morphology QTL
Source: PLoS Genet. 2024 Apr 25;20(4):e1011248. doi: 10.1371/journal.pgen.1011248 (PMC11075906; doi:10.1371/journal.pgen.1011248)

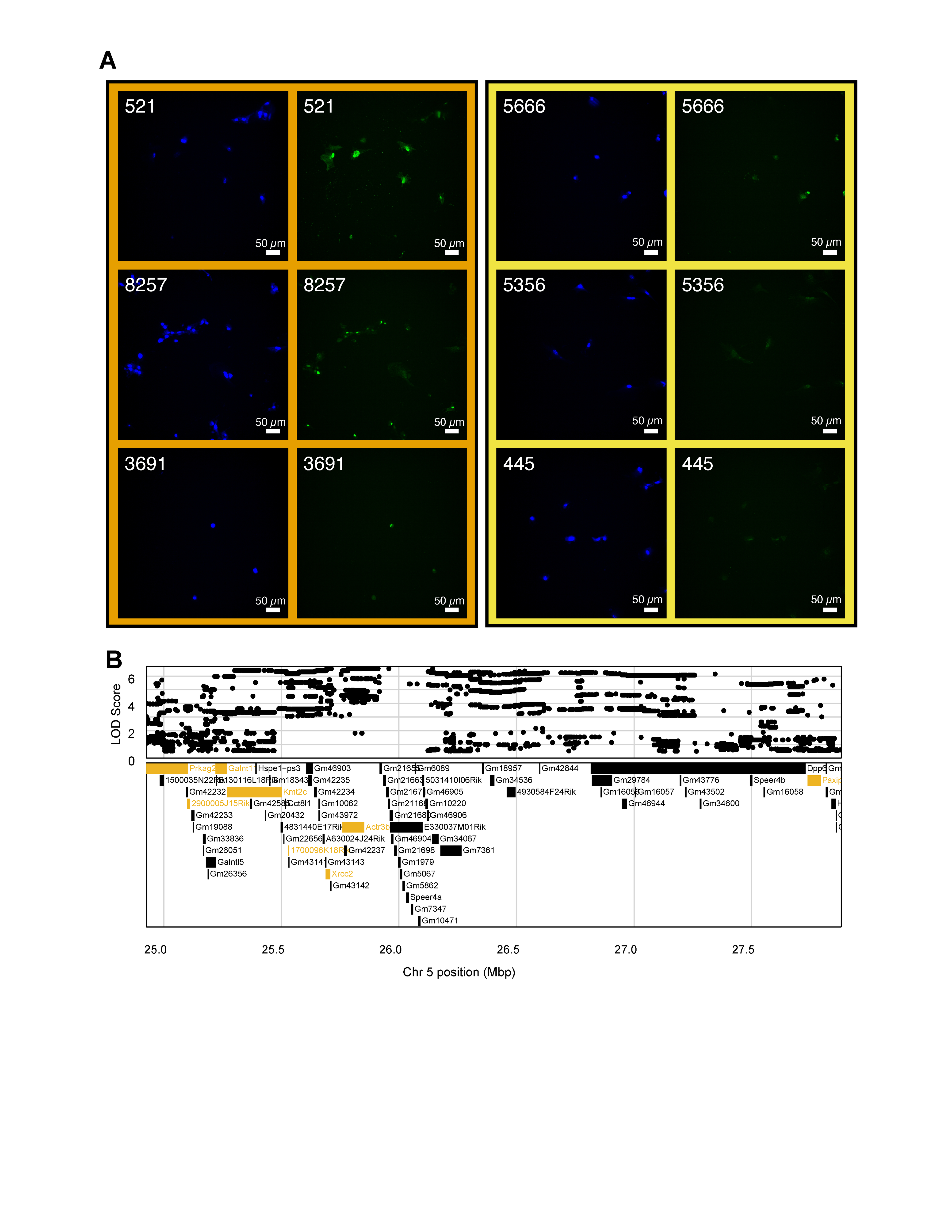

Supplement: S1 Fig — (A) Representative images for the two fibroblast lines at a 1.0 μM MMAIII concentration with nuclei labeled by Hoechst 33342 (blue) and γH2AX (Alexa-488 secondary; green) for primary fibroblasts with a 129 allele (orange; n = 3) versus an AJ/B6 allele (yellow; n = 3) at the maximum position for the EC90 nucleus Hoechst distribution texture hole (`EC90 Hoechst Nucleus Symmetry (02) Hole Mean per Well') cmQTL. (B) Variant association mapping within the CI the cmQTL EC90 nucleus Hoechst distribution texture hole (`EC90 Hoechst Nucleus Symmetry (02) Hole Mean per Well'). Top panel shows the LOD scores of the known, segregating variants in the 8 DO founders (GRCm38). Bottom panel shows the gene models within the respective CI. Each point represents a variant. Colors indicate whether a gene is expressed > 0.5 TPM (gold) or < 0.5 TPM (black). (TIF) [file pgen.1011248.s001.tif]

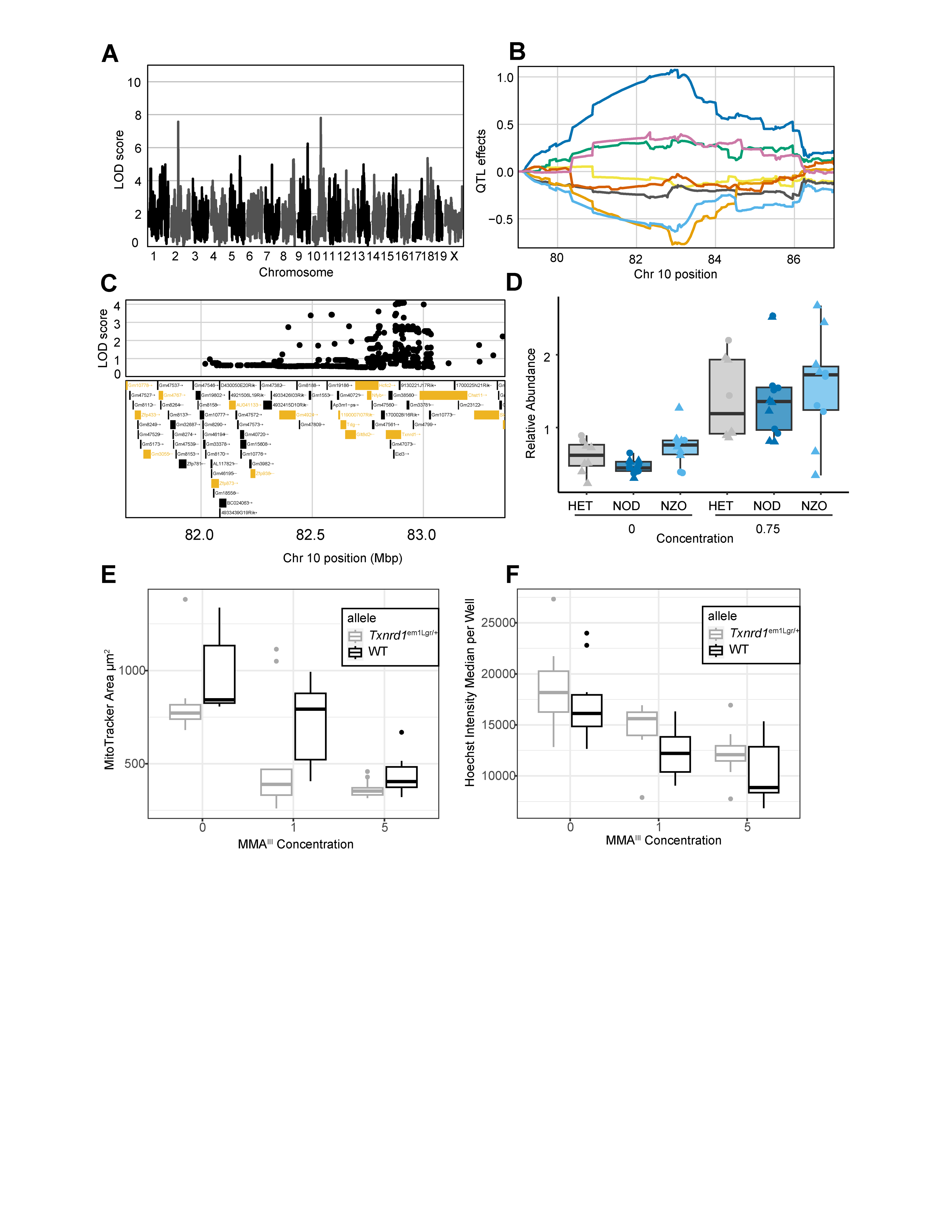

Supplement: S2 Fig — (A) QTL scan for the EC90 cell MitoTracker distribution (EC90_nonborder_mitosmooth_symmetry) cmQTL with the maximum peak at chromosome 10:82,967,807 bp (GRCm38) and a LOD score of 7.64. (B) Haplotype effects plot showing the eight DO founders (colors, see Methods) for the EC90 cell MitoTracker distribution (EC90_nonborder_mitosmooth_symmetry) cmQTL across the surrounding region on chromosome 10 (Mbp). (C) Variant association mapping within the CI the cmQTL `γH2AX-negative cells slope Cell Area μm2 mean per well'. Top panel shows the LOD scores of the known, segregating variants in the 8 DO founders (GRCm38). Bottom panel shows the gene models within the respective CI. Each point represents a variant. Colors indicate whether a gene is expressed > 0.5 TPM (gold) or < 0.5 TPM (black). The arrow indicates the direction of transcription. (D) Relative abundance of TXNRD1 compared between DO fibroblast lines with NOD (n = 6), NZO (n = 5), and NOD/NZO (n = 4) alleles at the chromosome 10 locus. (E) MitoTracker Deep Red Cell Area across increasing MMAIII concentration for Txnrd1em1Lgr/+ (n = 3) compared to B6 control (n = 3) primary fibroblasts. Colors indicate wild-type (black) compared to Txnrd1em1Lgr/+ (gray) primary fibroblast lines. (F) `Hoechst 33342 intensity' across increasing MMAIII concentration for Txnrd1em1Lgr/+ (n = 3) compared to B6 control (n = 3) primary fibroblasts. Colors indicate wild-type (black) compared to Txnrd1em1Lgr/+ (gray) primary fibroblast lines. (TIF) [file pgen.1011248.s002.tif]

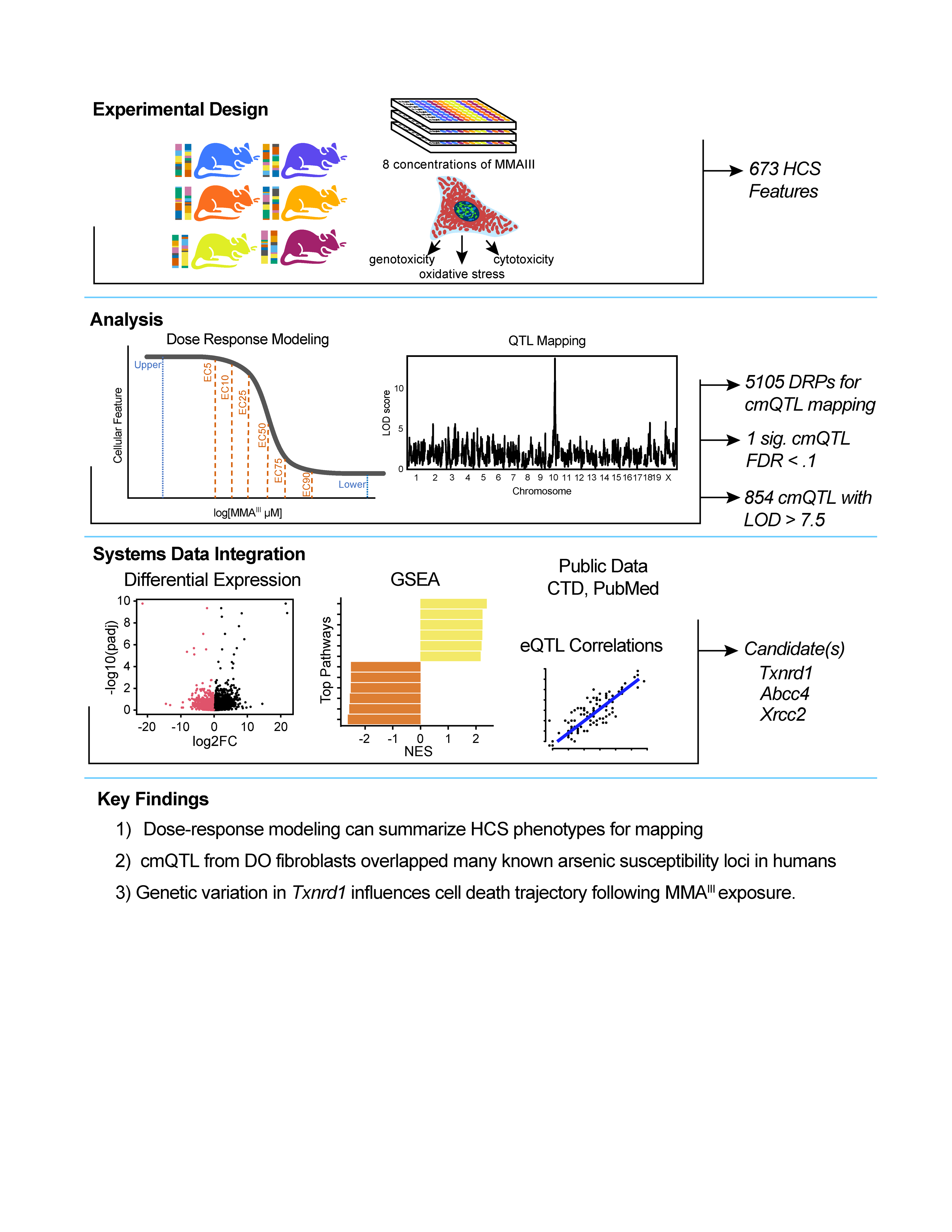

Supplement: S3 Fig — Primary fibroblasts were derived from Diversity Outbred (DO) mice and were exposed to 8 increasing concentrations of MMAIII. They were labeled with Hoechst 33342, MitoTracker Deep Red, and γH2AX/488, imaged using the Operetta (PerkinElmer) at 20X, and images were analyzed using Harmony 4.9 to yield 673 HCS cellular features. These cellular features were then fit to a log-logistic dose-response model where parameters were extracted including the starting asymptote, slope, EC5, EC10, EC25, EC50, EC75, EC90, and maximum asymptote to yield. Following linear mixed modeling summarization of replicates and inter-plate batch correction, 5105 cellular traits remained for cmQTL mapping. Whole genome scans were performed across all 5105 cellular features to identify cellular morphology quantitative trait loci (QTL) influencing fibroblast sensitivity to MMAIII where 1 cmQTL reached significance (FDR ≤ .1) and 854 cmQTLs had suggestive LOD scores > 7.5. Differential expression, gene set enrichment, previous gene-arsenical interactions curated from the Comparative Toxicogenomics Database (CTD), and correlated haplotype effects between cmQTL and DO eQTL data across many tissues were used to nominate candidate genes and variants. (TIF) [file pgen.1011248.s003.tif]
